# Intelligence Sparse Sensor Network for Automatic Early Evaluation of General Movements in Infants

**DOI:** 10.1002/advs.202306025

**Published:** 2024-03-06

**Authors:** Benkun Bao, Senhao Zhang, Honghua Li, Weidong Cui, Kai Guo, Yingying Zhang, Kerong Yang, Shuai Liu, Yao Tong, Jia Zhu, Yuan Lin, Huanlan Xu, Hongbo Yang, Xiankai Cheng, Huanyu Cheng

**Affiliations:** ^1^ School of Biomedical Engineering (Suzhou) Division of Life Sciences and Medicine University of Science and Technology of China Hefei 230022 P. R. China; ^2^ Suzhou Institute of Biomedical Engineering and Technology Chinese Academy of Science Suzhou 215011 P. R. China; ^3^ Department of Engineering Science and Mechanics The Pennsylvania State University University Park PA 16802 USA; ^4^ Department of Developmental and Behavioral Pediatrics The First Hospital of Jilin University Changchun 130021 P. R. China; ^5^ School of Material and Energy University of Electronic Science and Technology of China Chengdu 610054 P. R. China; ^6^ Department of Rehabilitation Medicine Children's Hospital of Soochow University Suzhou 215025 P. R. China

**Keywords:** assessment of general movements, soft wireless IMU devices, tiny machine learning algorithm for automatic early evaluation, wearable sparse sensor network

## Abstract

General movements (GMs) have been widely used for the early clinical evaluation of infant brain development, allowing immediate evaluation of potential development disorders and timely rehabilitation. The infants’ general movements can be captured digitally, but the lack of quantitative assessment and well‐trained clinical pediatricians presents an obstacle for many years to achieve wider deployment, especially in low‐resource settings. There is a high potential to explore wearable sensors for movement analysis due to outstanding privacy, low cost, and easy‐to‐use features. This work presents a sparse sensor network with soft wireless IMU devices (SWDs) for automatic early evaluation of general movements in infants. The sparse network consisting of only five sensor nodes (SWDs) with robust mechanical properties and excellent biocompatibility continuously and stably captures full‐body motion data. The proof‐of‐the‐concept clinical testing with 23 infants showcases outstanding performance in recognizing neonatal activities, confirming the reliability of the system. Taken together with a tiny machine learning algorithm, the system can automatically identify risky infants based on the GMs, with an accuracy of up to 100% (99.9%). The wearable sparse sensor network with an artificial intelligence‐based algorithm facilitates intelligent evaluation of infant brain development and early diagnosis of development disorders.

## Graphical abstract

This work presents a machine learning‐powered wireless sparse sensor network with soft wireless inertial motion units (IMUs) devices for automatic evaluation of general movements (GMs) in infants. The proof‐of‐the‐concept clinical testing demonstrates the system can automatically and reliably identify risky infants based on the GMs with an accuracy of up to 100%, indicating its potential as an intelligent evaluation tool for infant brain development disorders.

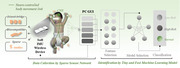

## 1 Introduction

Brain development is a process of continuous remodeling during the whole infant development stage.^[^ ^1^ ^]^ Early intervention may improve outcomes in developed neuromotor diseases, but this requires accurate early identification of risky infants.^[^ ^2^ ^]^ Functional brain imaging (i.e., MRI,^[^ ^3^ ^]^ fNIRs) can directly identify intracranial structural lesions in high‐risk children and predict neurodevelopmental diseases, especially cerebral palsy^[^ ^4^ ^]^ (CP). However, expensive equipment, the requirement for skilled professionals, and the complex imaging process present challenges for use in large‐scale early detection, especially in remote and low‐resource settings.^[^ ^5^ ^]^ Fortunately, clinical data indicate that infant movement behaviors can reveal rich information about the development of the central nervous system.^[^ ^6^, ^7^ ^]^ For instance, motor development assessment methods^[^ ^8^ ^]^ (GMA, TIMP, HINE) are widely used based on some motor development scales^[^ ^9^ ^]^ (i.e., GDS, BSID, AIMS, PDMS) obtained from visual examination by specialized experts and clinicians in practice.^[^ ^10^ ^]^ Although GMA can predict CP with high sensitivity and specificity,^[^ ^11^ ^]^ it is not widely adopted clinically due to subjective judgments and the significantly lower number of clinicians specifically trained with Prechtl's GMA method.^[^ ^12^ ^]^ A new digital approach (or digitization of scales) is therefore urgently needed for the early evaluation of general movements in infants for clinical application.

The general movements of infants can also be captured by using a video camera.^[^ ^10^ ^]^ The spatial and temporal resolution of the video‐based system (i.e., multiview imagery, depth imagery, single RGB stream) is sufficient for movement analysis, but the data are inevitably accompanied by background noise.^[^ ^13^ ^]^ The large collected data also present challenges for high‐speed processing and efficient automatic classification.^[^ ^14^ ^]^ Besides privacy concerns, the complex camera setup and susceptibility to surrounding environments such as lighting further limit their real‐world implementation in community clinics or home settings.^[^ ^15^, ^16^, ^17^ ^]^ The camera's limited field of view makes it challenging to evaluate large‐area spatial posture and the engagements between children and parents or clinic workers are impeded^[^ ^18^ ^]^ as well. On the other hand, wearable sensors such as accelerometers,^[^ ^19^, ^20^ ^]^ and EMG electrodes^[^ ^21^ ^]^ can also be explored for general movements. To significantly reduce the risk of iatrogenic skin injuries due to the fragility associated with infants’ immature skin,^[^ ^22^ ^]^ it is highly desirable to explore soft wearable wireless devices with “skin‐like” mechanical properties^[^ ^23^, ^24^ ^]^ to interface with the neonatal skin. Despite recent advances in this class of soft devices^[^ ^25^ ^]^ to monitor various biomedical signals (e.g., acceleration/velocity,^[^ ^26^ ^]^ ECG,^[^ ^27^ ^]^ PPG,^[^ ^28^ ^]^ and cerebral hemodynamic^[^ ^29^ ^]^), challenges still exist for the automatic early evaluation of general movements in infants. Without losing detection accuracy, sparse sensor nodes in the soft wearable device^[^ ^30^, ^31^ ^]^ are preferred to reduce the impact of foreign body sensation and infants’ autonomous movement, as well as to cut the cost. Different from large‐size AI frameworks, tiny machine learning algorithms could provide rapid classification, but their use for accurate automatic detection at different network conditions still remains elusive.

**Figure 1:**
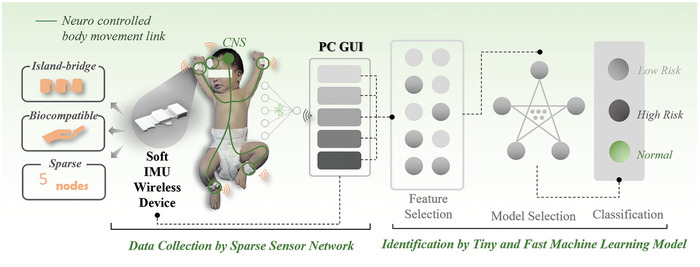
The schematic illustrating the system design and application of the intelligent sparse sensor network with soft wireless IMU devices (SSN‐SWDs).

Herein, this work presents a machine learning‐powered wireless sparse sensor network with soft wireless inertial motion units (IMUs) devices for automatic early evaluation of general movements (GMs) in infants (**Figure** **1**). In particular, five IMU sensors with biocompatible soft materials are connected to the BLE (Bluetooth low energy) one‐to‐five technology unit by serpentine structures, forming a wireless sparse sensor network to allow for comfortable contact with the skin of infants and a robust collection of movement data. The data streams generated by this sensor network are processed by a tiny machine‐learning algorithm with a custom‐developed graphical user interface (GUI) for the automatic identification of infants at risk of abnormal neural development. The proof‐of‐the‐concept pilot study of validating the device on infants between 4 and 21 weeks of age (*n =* 23) in low‐resource (Quwo, Linfen in China) settings demonstrates the practical utility of the device for the assessment of general movements and identification of infants at risk.

## 2 Results and Discussion

### 2.1 Sparse Sensor Network with Soft Wireless IMU Devices (SSN‐SWDs)

#### 2.1.1 SWD Design Layouts and Sensor Network Configuration

**Figure 2:**
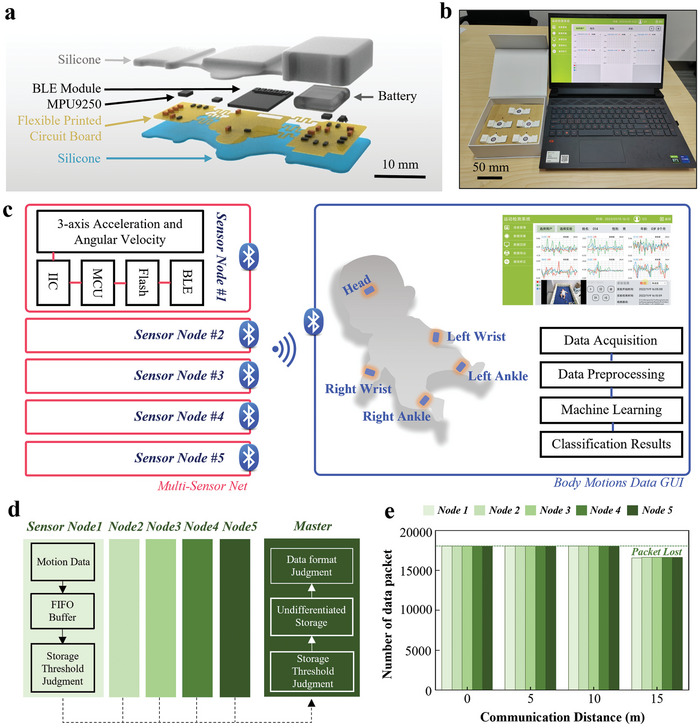
Design and application of the intelligent sparse sensor network with soft wireless IMU devices (SSN‐SWDs) for automatic early evaluation of general movements (GMs) in infants. a) Exploded view showing the structure of the SWD comprising of electronic components and flexible printed circuit board encapsulated by soft silicone elastomers. b) Photograph of the fully integrated SSN‐SWDs system that includes five time‐synchronized SWDs and a graphical user interface (GUI) on a desktop. c) Functional block diagram of the sparse sensor network showing the components of the SWD: 3‐axis acceleration and angular velocity sensor, IIC interface, MCU, flash memory, and radio interface, with a self‐developed GUI that includes data acquisition and analysis modules. d) Flowchart of the time‐synchronized strategy. e) Measured numbers of data packets with sensor nodes positioned at different distances from the GUI.

The soft wireless IMU device (SWD) with the serpentine structure design and soft biocompatible silicone can conform to the delicate skin of infants with a medical‐grade silicone adhesive, allowing for the real‐time monitoring of general movements through 3‐axis acceleration and angular velocity (**Figure** **2**a). The device consists of three main units on a flexible printed circuit board (fPCB) interconnected by serpentine traces: 1) an IMU for continuous monitoring of movement; 2) a low‐power wireless communication unit based on BLE; and 3) a power management unit based on a lithium polymer battery to support more than 4 h of continuous real‐time data streaming (Figure S1, Supporting Information). The resulting fPCB encapsulated with a medical‐grade silicone elastomer yields a SWD with a light weight of 4.1 g and a small footprint of 54× 27 × 4 mm (Figure S2a, Supporting Information). The charging unit with magnetic pogo pins fabricated by 3D printing allows for electrical and mechanical coupling to the SWD for stable and fast charging (Figure S2b, Supporting Information).

Although rich sensing nodes can provide a high spatial resolution of movement information, they are associated with a clear foreign body sensation and inevitably affect the autonomous movement of infants. Emphasis has been given to the movement of limbs and the head by experienced clinicians when it comes to assessing infants’ GMs. Therefore, the sparse sensor network in this work strategically places five physically separated but wirelessly connected SWDs on the forehead, wrists, and ankles of the infants (Figure 2b). Specifically, the captured raw data of 3‐axis acceleration and angular velocity in hexadecimal format can be stored by the micro control unit (MCU) in the Flash buffer (Figure 2c). The wireless connection is made possible by the BLE one‐to‐five technology, with the block diagrams showing the design architecture and overall data flow in Figure 2d. The custom constant frequency of each sensor node set by a 32.768 kHz clock timer allows periodic readings of the data with a given length. Once the data length exceeds the custom threshold (24 bytes), the raw data will be broadcast to the computer by the BLE module. Moreover, the custom synchronization strategy exploits a 2.4 GHz wireless transmission scheme in a one‐to‐five technology, allowing for communication of the time‐synchronized data. The GUI on the computer then stores and parses these data with a defined length with a custom protocol to display the movement data from each sensor node. The integrated system allows the wireless operation of the sensor network that is physically separated from the computer at a working distance of ≈10 m without packet loss (Figure 2e). The high accuracy of the analysis, classification, and evaluation of general movements in infants via the GUI showcases the feasibility of the integrated system for early diagnosis of developmental disorders.

#### 2.1.2 Mechanical Property and Biocompatibility of the SWD

**Figure 3:**
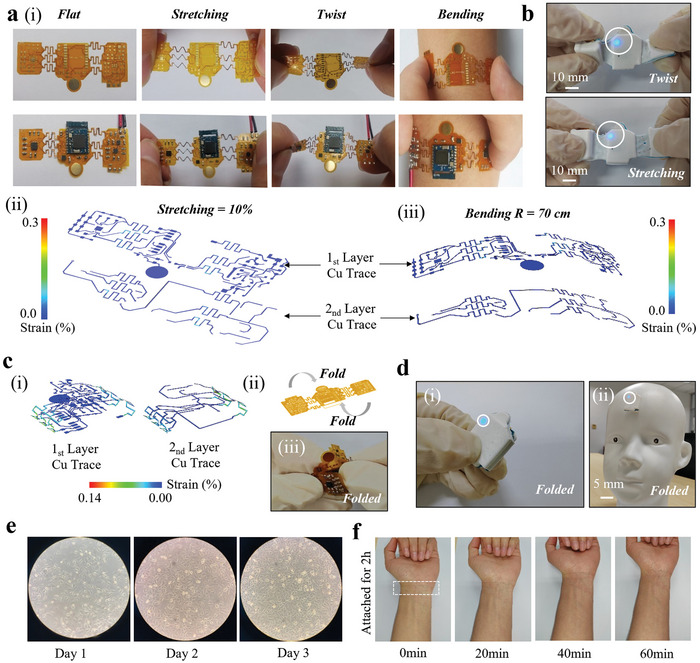
Mechanical property and biocompatibility of the SWD. a‐i) Optical images of the bare fPCB during various mechanical deformations and the corresponding finite element analysis of the fPCB upon (ii) stretching (10%) and (iii) bending (*R* = 70 cm). b) Optical images of the encapsulated SWD upon twisting and stretching (the LED light is on to indicate the working condition). c‐i) The corresponding finite element analysis, (ii) schematic diagram, and (iii) optical image to show the folding of the fPCB for minimized footprint. d‐i) Optical image of the folded encapsulated SWD and (ii) its placement on the forehead of a neonatal head lime model. e) Optical microscopy images of the human lung fibroblast cells cultured with Ecoflex 0030 and Gel 4317 for 1, 2, and 3 d. f) Optical images of the skin after peeling off the SWD (on the skin for 2 h) to show no skin irritation.

The combination of miniaturized commercial off‐the‐shelf (COTS) components with soft materials and stretchable structures could provide the hybrid electronics with a soft and biocompatible interface to the delicate neonatal skin for robust continuous recording. The fPCB interconnected by serpentine‐shaped conductive traces allows the device to withstand mechanical deformations (e.g., stretching, twisting, and bending) without mechanical failure (**Figure** **3**a). The serpentine trace with optimized structures results in a maximum principal strain in the PI/Cu layer being less than the yield strain (0.3%) under various mechanical loads. The system is configured to turn on the LED light only when the establishment of a Bluetooth low energy (BLE) connection is successful (Figure S3, Supporting Information). Therefore, the LED indicator of the Bluetooth remains ON to indicate robust performance during stretching and twisting (Figure 3b and Movie S1, Supporting Information). Considering the limited size of the infant's forehead, it is vital to exploit the device with a minimized footprint. Although multi‐layer PCB design could be explored to reduce the size, it would result in significantly increased manufacturing costs. As an alternative, folding of the three fPCBs at the serpentine region reduces the device footprint to 1.6 × 2 cm^2^ with a maximum strain of 0.25% in Cu (Figure 3c and Movie S2, Supporting Information). The folded device size is much smaller than the flat area of 4.5 × 3 cm^2^ at 18 weeks of gestational age.^[^ ^32^ ^]^ The device function is not affected by folding as the Bluetooth indicator remains lit on the infant model's forehead (Figure 3d). Potential skin injury has been a significant concern in the standard clinical care for infants. The cytocompatibility test by culturing human lung adenocarcinoma cell line A549^[^ ^33^ ^]^ with Ecoflex 0030 for 2 d demonstrates healthy growth of the cells and biocompatibility of the device materials (Figure 3e). Furthermore, there is no sign of skin irritation or allergic reactions after using the device on the skin for 2 h (Figure 3f and Figure S4, Supporting Information). With the drastically reduced cost in manufacturing, the device cost has been reduced to $15 per sensor node. Together with the excellent biocompatibility and high accuracy for automatic detection, the integrated device from this work compares favorably with the commercial equipment and other reported works (Tables S1 and S2, Supporting Information). The low‐cost yet high‐fidelity device promises wide adoption for large‐scale screening of infants at risk especially in regions with limited medical resources.

#### 2.1.3 Characterization of the Electrical Property

The 3‐axis acceleration (Acc.) and angular velocity (Av.) captured by the IMU in the integrated device from different body positions can be directly compared to those from the commercial rigid IMU sensor by using the Pearson correlation coefficient^[^ ^34^ ^]^ (*r*), defined as:

(1) $$r\;=\;\frac{\sum_{i\;=\;1}^{n}\left(X_{i}-\bar{X}\right)\left(Y_{i}-\bar{Y}\right)}{\sqrt{\sum_{i\;=\;1}^{n}{\left(X_{i}-\bar{X}\right)}^{2}}\sqrt{\sum_{i\;=\;1}^{n}{\left(Y_{i}-\bar{Y}\right)}^{2}}}$$

**Figure 4:**
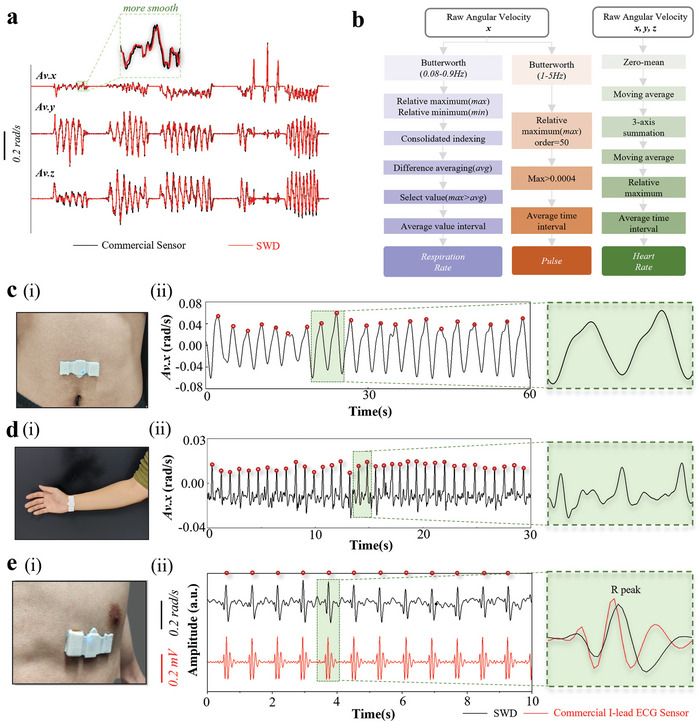
The electrical property of the SWD and its potential use for physiological monitoring. a) Comparison of the normalized signals simultaneously acquired from the 3‐axis commercial accelerometer (black line) and our device (red line) at the same position. b) Block diagram of the post‐processing algorithms to obtain the respiration, pulse, and heart rates. Signals detected by the device placed on the c) abdomen for respiration, d) wrist for pulse wave, and e) central anterior region for the heartbeat, with detected peaks used for calculating the respiration, pulse, and heart rates. The mechano‐acoustic signal captured by our device (black line) is also compared with the ECG signals (red line) recorded by the commercial I‐lead equipment.

The 3‐axis angular velocity from the device matches remarkably well with that obtained from a commercial rigid sensor (PN3 pro, Noitom) (**Figure** **4**a), which exhibits *r* of 98.6%, 98.2%, and 97.7% for the *x‐, y‐*, and *z*‐axis angular velocity between the two. Compared with the commercial rigid device, the soft integrated device is immune to the relative motion or motion artifacts for both high‐ and low‐frequency movements, because of the robust and stable sensor/skin interface during movement (Figure S5, Supporting Information). For instance, the stable/intimate contact can reduce the impact of the relative motion at the interface on the measured data and calculated feature values for the machine learning model (Figure S6, Supporting Information). The on‐chip low‐pass filter can also help remove the background noise to enhance the accuracy of the captured movements for clinical evaluation of GMs. In addition, subtle human physiological information such as respiration rate (RR), pulse rate, and heart rate (HR) can be obtained from the 3‐axis raw angular velocity based on the data processing flow algorithms shown in Figure 4b. In brief, the *x‐*axis angular velocity measured with the device placed on the abdomen gives the respiration signal after applying a bandpass filter (low cutoff frequency *f*_low_ of 0.08 Hz and high cutoff frequency *f*_high_ of 0.9 Hz) and a custom peak‐detection algorithm (Figure 4c and Note S1, Supporting Information). The pulse wave is separated from the raw *x‐*axis angular velocity measured from the wrist by modifying the bandpass filter (*f_low_* = 1 Hz, *f*_high_ = 5 Hz) and the custom algorithm (Figure 4d and Note S2, Supporting Information). Normalizing and summing the 3‐axis angular velocities measured near the heart generates a heart waveform that correlates well with the waveform from the commercial I‐lead electrocardiogram (ECG) equipment (Figure 4e). The easy detection of the R peak using a custom peak‐detection algorithm gives the heart rate (Note S3, Supporting Information).

### 2.2 Automatic Early Evaluation of GMs in Infants Based on Tiny ML Module

#### 2.2.1 Clinical Experimental Setup and Data Collection

**Figure 5:**
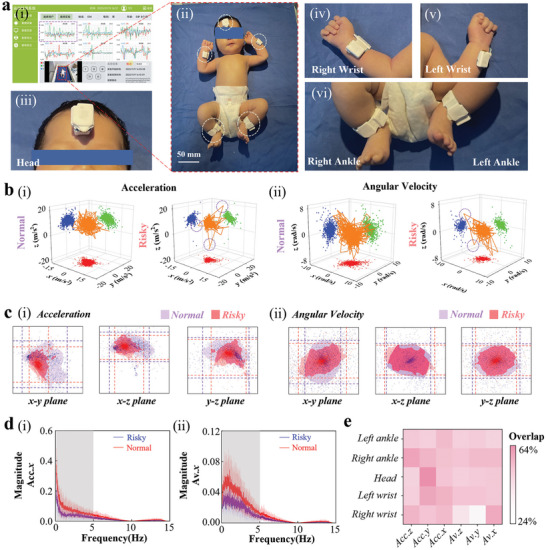
Clinical configuration for dataset building, visualization, and pre‐analysis. a) Photograph showing the experimental setup with the integrated device placed on the right wrist, left wrist, right ankle, left ankle, and the head of an infant (12 week) lying in a crib. b) 3D scatter plots from 3‐axis (i) acceleration and (ii) angular velocity on the left ankle of a “Normal” (left, ID‐01) and “Risky” infant (right, ID‐20). c) The distribution of projected scatter maps of (i) acceleration and (ii) angular velocity on *x*‐*y*, *x*‐*z*, and *y*‐*z* planes based on the distribution density of the “Normal” and “Risky” samples larger than 0.3%. d) The power spectrum of the x‐axis acceleration (Acc.x) and angular velocity (Av.x) with error band from the left ankle (red/blue: “Normal”/“Risky” infant) (*n* = 18). e) Heatmaps of overlap frequency rates of 3‐axis acceleration and angular velocity between the “Normal” and “Risky” samples at different positions (*n* = 18).

To validate the automatic early evaluation of GMs in infants with the integrated device and tiny machine learning module, a standalone room that meets the requirements of clinical GMs assessment^[^ ^35^ ^]^ (Figure S7, Supporting Information) is used for the data collection. Although the integrated device does not pose a specific requirement for the room, the quiet room with no other stimuli (sound, light, etc.) is still used to capture the neonatal autonomous movement for clinical evaluation. The clinical evaluation system includes a video camera for real‐time recording, which is meticulously assessed and labeled by a team of three professional pediatricians from the First Hospital of Jilin University (Figure S7, Supporting Information). The SSN‐SWDs are also placed on the right wrist, left wrist, right ankle, left ankle, and the head of an infant lying in a crib, with minimal effect on the movement as shown in the insets (**Figure** **5**a). The collected data from both the camera and our device are stored and displayed by the custom GUI on the computer (Figure S8, Supporting Information).

In the proof‐of‐the‐concept pilot study, acceleration and angular velocity are continuously recorded for approximately 5 min from 23 neonatal subjects. Because the clinical evaluation of the GMs with the camera requires no crying in the infants, five samples with crying during the 5‐min data collection cannot be used. As a result, there are 12 infant samples labeled as “Normal”, and 6 labeled as “Risky” (3 “Low Risk” and 3 “High Risk”) (Table S3, Supporting Information), providing a labeled dataset for data processing, feature extraction, and training of machine learning models.

#### 2.2.2 Visualization and Pre‐analysis of the Dataset

In the clinical evaluation, normal infant subjects are associated with general movements that have a variable sequence, amplitude, speed, and intensity, with slight variations in the direction, rotation, and frequency of motion, exhibiting relatively smooth yet complex features.^[^ ^36^ ^]^ In comparison, the infants at risk often show the absence of GMs or abnormal GMs depending on the severity of the disorder.^[^ ^37^ ^]^ Therefore, it is possible to characterize the intensity and rotations of the body movement with the measured acceleration and angular velocity. The features could be analyzed and displayed in both time and frequency domains.

Compared to the “Normal” infant, the “Risky” subject exhibits a single abrupt change occasionally in the 3D line graphs of real‐time acceleration and angular velocity measured from the left ankle, resulting in a distinct separate bulge marked by the dotted circle (Figure 5b). The ranges of the measured acceleration and angular velocity from the “Risky” subject are also narrower than those from the “Normal” infant, which can be clearly observed in the projected scatter maps on different planes (Figure 5c). The range of acceleration from the “Normal” infant is approximately 2.5, 2, and 1.5 times larger than that of the “Risky” sample in the *x‐y*, *x‐z*, and *y‐z* planes, representatively, implying larger variability in the acceleration for less convulsive movements. This observation remains valid from all five sensing nodes as shown in the 2D line graphs of acceleration and angular velocity (Figure S9, Supporting Information).

In addition to the time domain, the power spectrum of acceleration and angular velocity in the frequency domain obtained by the fast Fourier transform (FFT) (Note S4, Supporting Information) also confirms the clear difference between the two groups (Figure 5d). The average power magnitude of the *x*‐axis acceleration (Acc*.x*) and angular velocity (Av*.x*) from the left ankle of all “Normal” samples exhibits a pronounced higher energy than that of “Risky” samples over the entire frequency range, confirming the higher variation in the movements of normal infants. The same characteristics are consistently reflected in the power spectrum of the *y*‐axis and *z*‐axis acceleration and angular velocity across all sensor nodes (Figures S10–S14, Supporting Information), which indicates that normal infants are associated with a higher variation in the movements across these sensor nodes. However, the error band (described in Note S5, Supporting Information) of these two groups overlaps, suggesting the challenge to just use this magnitude to differentiate the individuals within the two groups. Furthermore, the degree of overlap (or similarity) of movement signals at different positions in the frequency domain can be obtained by averaging the ratios of the coverage magnitude over the total magnitude at each frequency point (Note S6, Supporting Information). Although varied, the degree of overlap clearly exists in the 3‐axis acceleration and angular velocity from five sensing nodes at different positions between the “Normal” and “Risky” samples (Figure 5e). The lowest degree of overlap of 24.26% is observed in the *y*‐axis angular velocity from the right wrist, whereas the one in the *x*‐axis acceleration from the head is as high as 63.60%. While the low similarity of the features could contribute to the easy differentiation of two groups, there is no single feature to distinguish all individuals. Therefore, it's desirable to exploit machine learning algorithms to consider multiple features (in time and frequency domains) as in the clinical evaluation of GMs for comprehensive assessment and accurate classification.

#### 2.2.3 Classification of GMs Assessment by Building Tiny Machine Learning Flow

**Figure 6:**
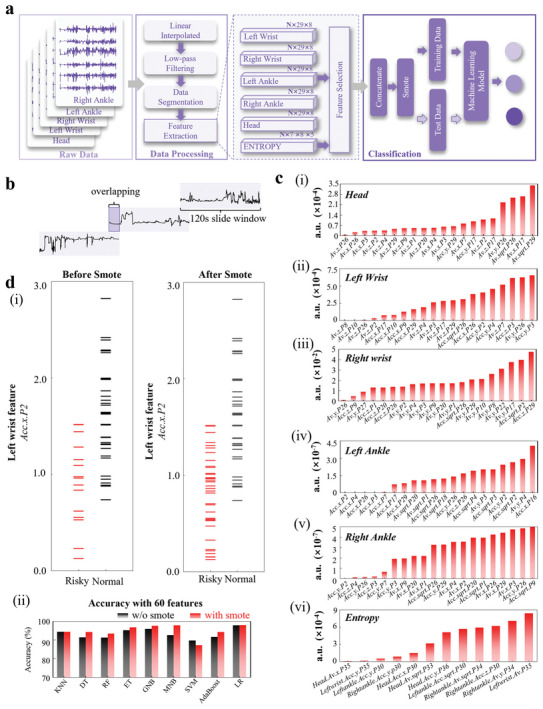
Strategies used for data processing and building a tiny machine learning model. a) The block diagram demonstrating the training of the machine‐learning model from data processing to classification. b) The use of long‐term overlapping sliding window processing for the raw data. c) The normalized *p*‐value of the (i–v) top 20 features from position feature matrices and (vi) top 12 features from the entropy feature matrix. (d‐i) The comparison in the feature (Acc.*x*.P2) distribution from the left wrist of the “Risky” and “Normal” samples before and after SMOTE, and (ii) the change in the classification accuracy from various machine learning models before and after SMOTE.

With the tiny machine learning model, the 3‐axis acceleration and angular velocity can be processed after data segmentation and feature extraction with high‐precision classification models to evaluate the neurological risks in infants (**Figure** **6**a). First, linear interpolation is explored to replace the missing values^[^ ^38^ ^]^ (“NULL”) for cleaning invalid data, and a Butterworth low‐pass filtering (*f*_high_ = 14 Hz) is used before data segmentation to filter out high‐frequency noise while retaining the neonatal movements^[^ ^39^ ^]^ The data stream is further segmented into frames with a length of 120 s including a 10 s overlapping length by using an overlapping sliding windows technique^[^ ^40^ ^]^ (Figure 6b), which gives over 90% different data in each segmented frame to ensure significant independence between data frames. As a result of the effective data segmentation, 54 data frames (38/16 with the “Normal”/“Risky” label) are obtained.

Adding the root mean square of the 3‐axis acceleration and angular velocity respectively expands the raw data from 6 to 8 dimensions^[^ ^41^ ^]^ (i.e., data fusion). With 29 distinct features in the time and frequency domains (Table S4, Supporting Information) that are effective for pattern recognition of general acceleration and angular velocity,^[^ ^42^ ^]^ the *N*×29×8 feature matrices (with *N* being the number of the samples) are formed for each sensing node. The seven entropy features (Table S4, Supporting Information) commonly employed to characterize the complexity of movement^[^ ^43^ ^]^ are also calculated, leading to the construction of an *N*×7×8×5 feature matrix that integrates seven entropy features derived from 8D data obtained from five distinct positions. As a result, six feature matrices are meticulously constructed to facilitate further feature selection.

Due to the significantly larger number of features than that of samples, it is important to perform feature selection. This can be done based on the *p*‐value that quantifies the statistical significance and contribution of each feature for the prediction. The *p*‐values were calculated with the above data (*n* = 54) using the ttest2 function (MATLAB, 2021) (see the details in the Experimental Section). Arranging elements within each feature matrix in ascending order based on their *p*‐values gives the top 20 features from the five position feature matrices and the top 12 features from the entropy feature matrix (Figure 6c and Note S7, Supporting Information). Furthermore, the best relative features at some sensor nodes only include one of acceleration or angular velocity according to the analysis of the *p*‐values. As a result, it is possible to further reduce fabrication cost and power consumption by replacing the current gyroscope with a 3‐axis gyroscope or removing dispensable data flow. For instance, the angular velocity exhibits a much larger impact than the acceleration at the neonatal head, which is attributed to the limited lateral shaking movement at the supine position. Equal‐weight feature processing^[^ ^44^ ^]^ is then employed to construct the new 60 features by integrating the top 10 features with a *p*‐value less than 0.05 from each feature matrix, which avoids overfitting of the model.^[^ ^45^ ^]^

As the number of the “Normal” samples is much larger than that of the “Risky” samples even after data segmentation, the accuracy of the prediction models can be greatly affected. To address this challenge, the synthetic minority oversampling technique^[^ ^46^ ^]^ (SMOTE) is utilized to generate new synthetic samples by interpolating samples in the minority class to balance the class distribution, which could enhance the performance and generalization of classification models. The comparison in the distribution of features before and after SMOTE indicates that the generated synthetic new data are within the original data distribution (Figure 6d‐i and Figure S15, Supporting Information). Further comparison in the accuracy of various machine learning models^[^ ^47^ ^]^ (except for SVM) to differentiate the “Risky” from “Normal” samples shows a clear enhancement in the accuracy after using SMOTE (Figure 6d‐ii and Tables S5 and S6, Supporting Information). The most significant improvement is observed for MNB, with its accuracy increased from 93.3% to 99.5%.

**Figure 7:**
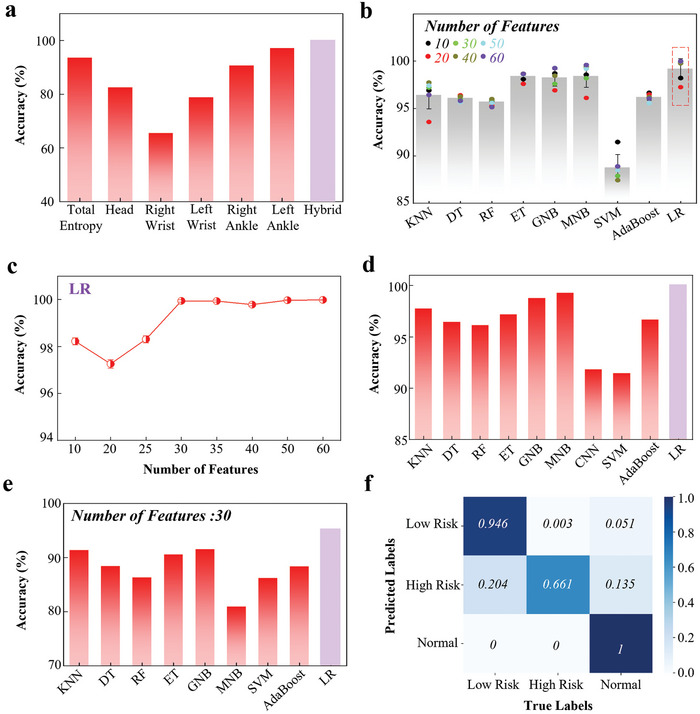
Automatic early evaluation of general movements in infants with the integrated device. The binary classification accuracy as a function of a) the applied various feature matrixes using the LR model and b) various machine learning models with different numbers of features. c) The binary classification accuracy versus the number of features and d) the accuracy of various machine learning models with 30 features. e) The three‐label classification accuracy of the various machine learning models with 30 features and f) the confusion matrix from the logistic regression model.

To further use the tiny machine learning model for rapid detection in low‐resource settings, there is a strong motivation to explore dimension reduction in the features to remove excessive redundancy in the prediction models. Although significantly deteriorated accuracy from 99.9% to 65.5% is observed for the data just from the right wrist with the logistic regression^[^ ^48^ ^]^ (LR) model, the accuracy from the left ankle is close to the optimum one obtained with hybrid features (**Figure** **7**a). Because the clinical general movement assessment relies on the entire body movement, the following investigations exploit hybrid features unless otherwise specified. The results also reveal the difference between feature matrices from different sensor nodes, so the weight for them should be different rather than the same. For instance, the feature matrix of the ankle should have a larger weight than that of the wrist. Similarly, the number of features from each matrix could be different. As the dimension of the feature matrix is reduced from 60 to 10, the changes in the accuracy from 9 distinct machine learning models^[^ ^49^ ^]^ are different (Figure 7b and Table S7, Supporting Information). The comparison leads to the choice of the LR model in the subsequent studies for its high average accuracy of 99.2%. There is minimal fluctuation in the binary classification accuracy as the dimension of the feature matrix is reduced from 60 to 30, maintaining a high level of 99.9% (Figure 7c). When 30 features are used for all models, the LR model clearly outperforms the others (Figure 7d).

Convolutional neural network (CNN), as a class of feedforward neural network with convolution computation and deep structure, is currently widely adopted for classification. After several rounds of training, a two‐layer CNN model (Figure S16, Supporting Information) can achieve an accuracy of 91.81% for the binary classification of GMs assessment (Figure 7d). Although the accuracy of CNNs may be constrained by the limited scale of the dataset in the current study, the intrinsic emphasis on data interaction within the network still suggests a high potential for the creation of more meaningful models as the data volume expands. However for this study, the LR model is still considered to be the most suitable classification model for the automatic evaluation of general movements from infants. Besides the binary classification, the samples with the “Normal,” “High Risk,” and “Low Risk” labels by the team of clinical experts (Table S3, Supporting Information) can also be classified but with lower accuracy for all models (Figure 7e). The LR model still exhibits a remarkable accuracy of 95.3%, outperforming the other models. The confusion matrix further indicates a high accuracy of 100% and 94.6% for the samples with “Normal” and “Low Risk” labels (Figure 7f). However, the accuracy pertaining to the “High Risk” samples is only 66.1%, primarily due to the limited number of six samples available in this class for training and evaluation. Nevertheless, the results establish the feasibility of combining the integrated device with a tiny machine‐learning model for the automatic classification of general movements in infants, paving the way for early evaluation and assessment of brain development.

## 3 Conclusion

In summary, soft sparse sensor nodes wirelessly connected by the BLE one‐to‐five technology could be pliably attached to the delicate neonatal skin for monitoring the general movement of the infants. The integrated device combined with the custom GUI and a tiny machine‐learning model provides continuous and robust monitoring and processing of the general movements to achieve a prediction accuracy of nearly 100% in clinical settings. Furthermore, the model with the feature dimension reduced by half still achieves almost unchanged accuracy, showing the high potential for future deployment in low‐resource settings. As the medical cloud database builds up with more samples added in the future, the design strategies from this work could promise AI‐powered intelligent medical services for other disease applications such as lung function and stroke balance assessments as well as assisting rehabilitation in remote settings.

## 4 Experimental Section

### Fabrication of Soft Wireless IMU Device

The self‐designed flexible printed circuit board (fPCB) was fabricated by a commercial vendor (Shenzhen HuiTai Electronic Technology Co.). All electronic components such as the inertial measurement unit (IMU) (MPU9250, InvenSense) and power management unit based on a 3.7 V lithium polymer battery (40 mAh, AngJie) with a voltage and current protection integrated circuit (BQ24040, Texas Instruments) were mounted on the board with a reflow soldering process. Customized firmware was debugged and loaded to the BLE system on a chip (SoC) (nRF52832, Nordic Semiconductor) by Keil 5.0. Low‐modulus elastomer (Ecoflex 0030, Smooth‐On) with color paste (Turquoise and Whit, SILC Pig) was used for the top and bottom encapsulation layers with shapes defined by aluminum molds, leaving only the switch and charging ports exposed. Conformal stable contact of the device with the skin was achieved by using an adhesive silicone layer (Gel 4317, SILBIONE RT). Specifically, the adhesive silicone mixed with a weight ratio of 1:1 on a release paper substrate was cured at 60 °C in an oven for 20 min. After cooling to room temperature, a CO_2_ laser system was unitized for cutting the shapes matched to the SWD before assembly.

### Development of the Computer GUI

The commercial software Unity 2020.3.41 was used to develop the GUI system. Various Unity components such as Unity Engine, Unity UI, and Unity 2D were utilized to implement the primary functions of the GUI. C# scripting was employed to implement the GUI and data processing. With the help of MySQL Connector/NET, the Unity project was connected to a MySQL database (version 8.0.17.0) to facilitate data storage, exportation, and other functions. The Unity software was used to receive the data collected from the sensors in the integrated device and the data collection process was also recorded and saved in video format (Figure S8, Supporting Information). Additionally, the magnetic field correction function in the software using the C# programming language could implement the IMU magnetic field's ellipsoid fitting algorithm to remove the influence of the magnetic field, resulting in improved precision of the angel calculation for other applications in future investigations.

A personal information file library was created for each subject, allowing for the creation of personalized rehabilitation training plans for each individual in the future. Following the instructions of the software, an account was registered by the user, with a proprietary account file that links all subject information under that account automatically generated by the software. As a result, personal information was secured to facilitate future large‐scale studies.

### Machine Learning and CNN Classification Model Building

A computer with an Intel Core i5‐11400 processor, 16GB of memory, an NVIDIA GeForce RTX 3060 GPU, and a Windows 10 operating system was utilized for the training and testing of machine learning models. The algorithms were implemented using the Python programming language. The TensorFlow‐GPU 2.4 deep learning framework was used for CNN.

The collected data were randomly divided into training and testing sets using a hold‐out method, with 80% allocated to the training set and 20% to the testing set. For the logistic regression model, 50 rounds of hold‐out training were used, with fivefold cross‐validation in each round to obtain objective final results. The algorithm was also run for more than 10 rounds and the average value of the results was calculated to ensure consistency in the results.

For the convolutional neural network (CNN), a two‐layer CNN classification network was designed using the Keras framework. The network consisted of the following layers: 1) the first convolutional layer with 128 channels of 2×2 convolution and ReLU activation function, 2) the second convolutional layer with 64 channels of 3×3 convolution and ReLU activation function, 3) a dropout layer (*P* = 0.5), 4) a 2×2 max pooling layer, 4) a flattened layer to convert the multidimensional tensor to a 1D vector, 5) a fully connected layer with 100 neurons and ReLU activation function, and 6) an output layer with two neurons and a softmax activation function for classifying the results. The Adam optimizer was used to train the CNN model. The hold‐out method was also used for the training of the CNN, with 50 rounds of training in each iteration. This iteration process was repeated for 10 cycles and the average of the evaluation results was calculated to ensure consistency and reliability.

The evaluation metrics included precision, recall, accuracy, and F1 defined as follows.

(2) $$\mathrm{Precision}\;=\frac{\mathrm{TP}}{\mathrm{TP}+\mathrm{FP}}\;$$

(3) $$\mathrm{Recall}\;=\;\frac{\mathrm{TP}}{\mathrm{TP}+\mathrm{FN}}$$

(4) $$\mathrm{Accuracy}\;=\;\frac{\mathrm{TP}+\mathrm{TN}}{\mathrm{TP}+\mathrm{FP}+\mathrm{TN}+\mathrm{FN}}$$

(5) $$F1\;=\;\frac{2\times \mathrm{precision}\times \mathrm{recall}}{\mathrm{precision}+\mathrm{recall}}$$

where TP is true for predicted as positive, FP is false for predicted as positive, TN is true for predicted as negative, and FN is false for predicted as negative.

### Characterization of Mechanical Properties and Biocompatibility—Finite Element Analysis (FEA)

The commercial software ABAQUS (ABAQUS Analysis User's Manual 2010, version 6.10) was used to calculate the strain distribution in the PI/Cu layer and the sensor upon stretching and bending. The tetrahedral elements (C3D10) were used to model both the PI/Cu and sensor components. In the simulation, the PI/Cu/PI/Cu/PI composite was sandwiched between two Ecoflex0030 layers. The model involved approximately 3 × 10^5^ elements and the mesh refinement was carried out to ensure convergence. The elastic modulus (*E*) and Poisson's ratio (*ν*) of the PI are 2500 kPa and 0.34, respectively. The elastic modulus (*E*) of Cu is 119 GPa and Poisson's ratio (*ν*) is 0.34. Ecoflex0030 is modeled as a hyperelastic solid with a bulk modulus (C10) of 0.0 08054 Pa, a shear modulus (C01) of 0.0 02013 Pa, and a third‐order elasticity constant (*D*1) of 2. The elastic modulus of 155/100/150 GPa and a Poisson's ratio of 0.255/0.25/0.3 were used for the chip/capacitor/resistor.

### Characterization of Mechanical Properties and Biocompatibility—In Vitro Evaluation of Cell Biocompatibility

A549 cells were cultured in the DMEM medium (Gibco, USA) with 10% fetal calf serum (CelliGent, New Zealand), 100 units/mL penicillin, 100 µg mL^−1^ streptomycin (Invitrogen, USA), and 3 g ecoflex0030. The culturing was maintained at 37 °C in a humidified 5% CO_2_ atmosphere. Cell survival was then observed using microscopy after 0, 24, and 48 h of cell culture.

### Ethical Approval and Experimental Design for Human Study

The human subject study was approved by the Medical Ethics Committee of Bethune First Hospital of Jilin University (Approval No. IRB202202‐001). After confirming that the subjects were awake and quiet and able to perform basic limb movements, the participants were recruited for the study. Prior to the experiment, the guardians of the participants were informed of the experimental details (e.g., the purpose, procedure, and other related information). The informed consent form was signed and a detailed information form about the participants was completed by the guardians.

The data were collected to assess infant movement and neurodevelopment only for clinical research. During the data collection from the sensor nodes for about 6 min, the video recording of the participants lying on their backs in a crib was also performed. A quiet environment was maintained during the experiment to avoid affecting the sensory perception of the participants. The acceleration and angular velocity were collected at a frequency of 30 Hz from the integrated device on the participants and the collected data were transmitted to the computer through low‐power Bluetooth in real time for display on the custom GUI. Confidentiality was strictly maintained, with the data made only available to the physicians. In all other use situations, the data were processed with personal information removed.

### Statistical Analysis

The unpaired T‐test was used to investigate dissimilarities between features in “Normal” and “Risky” samples (*n* = 54). Specifically, the ttest2 function (MATLAB 2021) was used to calculate the *p*‐value for each feature in “Normal” and “Risky” samples. A significance threshold of 0.05 was applied, and features with *p*‐values below this threshold were considered statistically significant. Subsequently, the features with *p*‐values less than 0.05 were sorted in each feature matrix based on their significance and contribution (Figure 6b). The optimized feature matrices were obtained for further feature selection and construction of tiny machine learning classification modules.

## Conflict of Interest

The authors declare no conflict of interest.

## Supporting information

Supporting Information

Supplemental Movie 1

Supplemental Movie 2

## Data Availability

The movement data from a total of 23 infants were collected in this study. The raw data were stored in .csv format. All personally identifiable information and sensitive data were removed and obfuscated to protect the privacy of the participants. The data supporting the results of this study are available within the paper and from the authors.
