# Supplementary material for: Intelligence Sparse Sensor Network for Automatic Early Evaluation of General Movements in Infants
Source: Adv Sci (Weinh). 2024 Mar 6;11(19):2306025. doi: 10.1002/advs.202306025 (PMC11109618; doi:10.1002/advs.202306025)
Supplement: Supplementary file 1 — Supporting Information [file ADVS-11-2306025-s001.pdf]

ADVANCED SCIENCE
Open Access

Supporting Information

for *Adv. Sci.*, DOI 10.1002/advs.202306025

Intelligence Sparse Sensor Network for Automatic Early Evaluation of General Movements in Infants

Benkun Bao, Senhao Zhang, Honghua Li, Weidong Cui, Kai Guo, Yingying Zhang, Kerong Yang, Shuai Liu, Yao Tong, Jia Zhu, Yuan Lin, Huanlan Xu, Hongbo Yang*, Xiankai Cheng* and Huanyu Cheng**

Supplementary Materials

Intelligence Sparse Sensor Network for Automatic Early Evaluation of General Movements in Infants

Benkun Bao[1,2§], Senhao Zhang[2,3§], Honghua Li[4], Weidong Cui[2], Kai Guo[1,2], Yingying Zhang[1,2], Kerong Yang[1,2], Shuai Liu[1,2], Yao Tong[1,2], Jia Zhu[5], Yuan Lin[5], Huanlan Xu[6], Hongbo Yang[1,2*], Xiankai Cheng[1,2*], Huanyu Cheng[3*]*

[1]School of Biomedical Engineering (Suzhou), Division of Life Sciences and Medicine, University of Science and Technology of China, Hefei, 230022, P.R. China

[2]Suzhou Institute of Biomedical Engineering and Technology, Chinese Academy of Science, Suzhou, 215011, P.R. China

[3]Department of Engineering Science and Mechanics, The Pennsylvania State University, University Park, 16802, USA

[4]Department of Developmental and Behavioral Pediatrics, The First Hospital of Jilin University, Changchun, 130021, P.R. China

[5]School of Material and Energy, University of Electronic Science and Technology of China, Chengdu 610054, P.R. China

[6]Department of Rehabilitation Medicine, Children's Hospital of Soochow University, Suzhou 215025, P.R. China

[§] These authors contributed equally to this work.
*To whom correspondence should be addressed. E-mail: zhangsh@sibet.ac.cn (S.Z.), yanghb@sibet.ac.cn (H.Y.), chengxk@sibet.ac.cn (X.C.), huanyu.cheng@psu.edu (H.C.)

Supplementary Note 1. Respiration rate acquisition algorithm

The acquired angular velocity data of the *x*-axis were filtered through a Butterworth bandpass filter[1] (low cut-off frequency f_{low} of 0.08 Hz and high cut-off frequency f_{high} of 0.9 Hz) to retain the breath information while removing redundant information. Next, the maximum and minimum values were searched and located, with the corresponding index values combined and retained. The difference between the neighboring maxima and minima larger than the threshold of 0.02 was identified as a respiratory event. Finally, the mean interval between the maximum values in the adjacent respiration events was used to determine the respiration rate.

Supplementary Note 2. Pulse acquisition algorithm

The acquired *x*-axis angular velocity was firstly filtered through a Butterworth bandpass filter (f_{low} = 1 Hz, f_{high} = 5 Hz). Next, a search was performed to locate the maximum value (neighborhood range = 50 sampling points). The time interval between the maximum values larger than the threshold of 0.0004 was used to obtain the pulse rate.

Supplementary Note 3. Heart rate acquisition algorithm

Firstly, the acquired angular velocity from each of the three axes (x-axis, y-axis, and z-axis) was processed for zero-mean to normalize the dataset. Next, a sliding mean process with a sliding window of five data points was applied to the processed data. The obtained data of the three axes were then summed according to the time synchronization, followed by another sliding mean process again with a sliding window of 20 data points. When the maximum value (neighborhood range = 10 sampling points) exceeded the threshold of 0.04, it was recorded as a valid maximum value. The average time interval between adjacent maximum values was used to calculate the heart rate.

Supplementary Note 4. Fast Fourier Transform of the motion data

The Discrete Fourier Transform (DFT) was performed to convert the acquired time-series motion data in discrete states from the sensors from the time to the frequency domain using the following equation:

$$\mathrm{F}(k)=\sum_{s=0}^{s\text{-}1}\mathrm{f}(s)w_s^{ks},\ \ k=0,1,2,3,4,\cdots,\mathrm{s}\text{-}1 \tag{1}$$

where $w_s=\mathrm{e}^{\frac{-2\pi\mathrm{j}}{\mathrm{s}}}$ is the rotation factor of the frequency domain response.

Based on the DFT, the Fast Fourier Transform (FFT) exploits the periodicity and symmetry of w_s and splits the original large-scale transform into several small-scale transforms, which is computationally faster and reduces computational efforts. The frequency from 0 to 0.1 Hz was removed to eliminate the influence of DC components. The maximum frequency was set to 15 Hz according to Nyquist's Law and the device sampling rate (30 samples per second).

Supplementary Note 5. Definition of the Error Band

The mean value ($\bar{X}$) and the standard deviation (SD) of N samples were calculated as:

$$\bar{X} = \frac{1}{N}\sum_{i=1}^{N} X_i \tag{2}$$

$$SD = \sqrt{\frac{1}{N-1}\sum_{i=1}^{N} (X_i - \bar{X})^2} \tag{3}$$

As a result, the upper and lower error points were obtained as follows:

$$\textbf{\textit{Upper error point}} = \bar{X} + SD \tag{4}$$

$$\textbf{\textit{Lower error point}} = \bar{X} - SD \tag{5}$$

The error band defined by these two limits represents the confidence interval of the samples.

Supplementary Note 6. Calculation of the overal degree of overlap

The coverage magnitude (***cov***) and the total magnitude (***tol***) at each frequency point were calculated as follows:

$$\boldsymbol{cov_i} = \min\left(\mathbf{mag\_n}_i^{\mathbf{max}}, \mathbf{mag\_r}_i^{\mathbf{max}}\right) - \max(\mathbf{mag\_n}_i^{\mathbf{min}}, \mathbf{mag\_r}_i^{\mathbf{min}}), \quad i=0, 1, 2, \cdots, \text{N-1} \qquad (6)$$

$$\boldsymbol{tol_i} = \max\left(\mathbf{mag\_n}_i^{\mathbf{max}}, \mathbf{mag\_r}_i^{\mathbf{max}}\right) - \min(\mathbf{mag\_n}_i^{\mathbf{min}}, \mathbf{mag\_r}_i^{\mathbf{min}}), \quad i=0, 1, 2, \cdots, \text{N-1} \qquad (7)$$

where $\mathbf{mag\_n}_i$ and $\mathbf{mag\_r}_i$ represent the power magnitude of normal and risky samples at the i_{th} frequency point, with the superscript max and min representing their maximum and minimum values. The overall **degree of overlap** (or similarity) was then calculated as follows:

$$\boldsymbol{degree\ of\ overlap} = \frac{\sum_{i=0}^{\text{N-1}} \frac{\mathbf{cov}_i}{\mathbf{tol}_i}}{\text{N}}, \quad i = 0, 1, 2, \cdots, \text{N-1}. \qquad (8)$$

Supplementary Note 7. Definition of the feature's abbreviations

(1) The representative feature "*Av.x.P1*" in the sensing node represents the mean value of the *x*-axis angular velocity, with the first term representing the raw data type (***Acc.*** and ***Av.*** for the acceleration and angular velocity respectively), the second term representing the dimension of the data (i.e., ***x.***, ***y.***, ***z.***, and ***sqrt.***), and the third term representing the value type (***P1 – P29*** **in Table S3**).

(2) The representative entropy feature "*Head.Av.x.P35*" represents the permutation entropy of the head x-axis angular velocity, with the first term representing the position of the sensor node (*Head, Leftwrist, Rightwrist, Leftankle, and Rightankle*), the second term representing the raw data type (***Acc.*** and ***Av.***), the third term representing the dimension of the data (***x.***, ***y.***, ***z.***, and ***sqrt.***), and the fourth term representing the value type (***P30 - P36*** in **Table S3**).

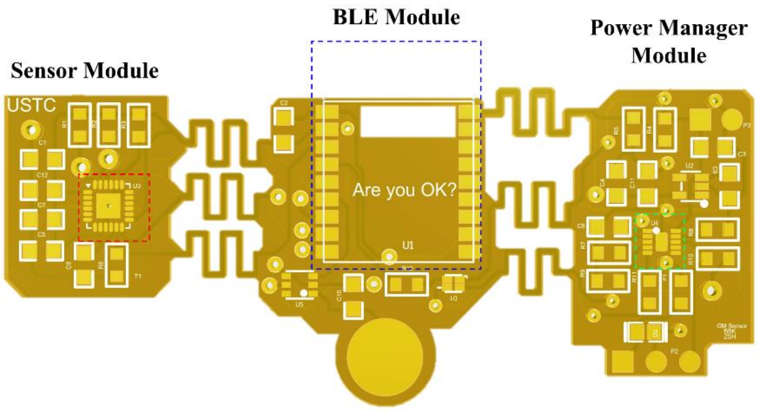

Figure S1. The overall layout of the circuit board for SWD.

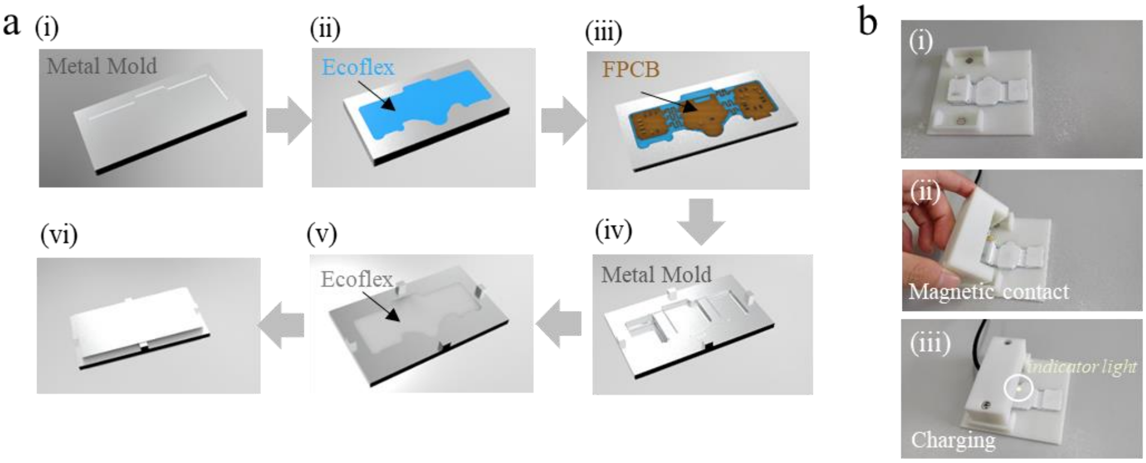

Figure S2. Encapsulation process and charging for SWD. (a) Schematic showing the encapsulation processing for SWD based on soft materials. (i) After preparing the metal mold with the bottom encapsulation layer, (ii) casting and curing Ecoflex0030 are followed by (iii) laminating and aligning the fPCB of SWD. (iv) After preparing the metal mold and (v) casting Ecoflex0030, (vi) assembling the two and curing the top encapsulation layer completes the encapsulation process. **(b)** Optical images showing the easy-to-use charging for SWD based on magnetic pogo pin.

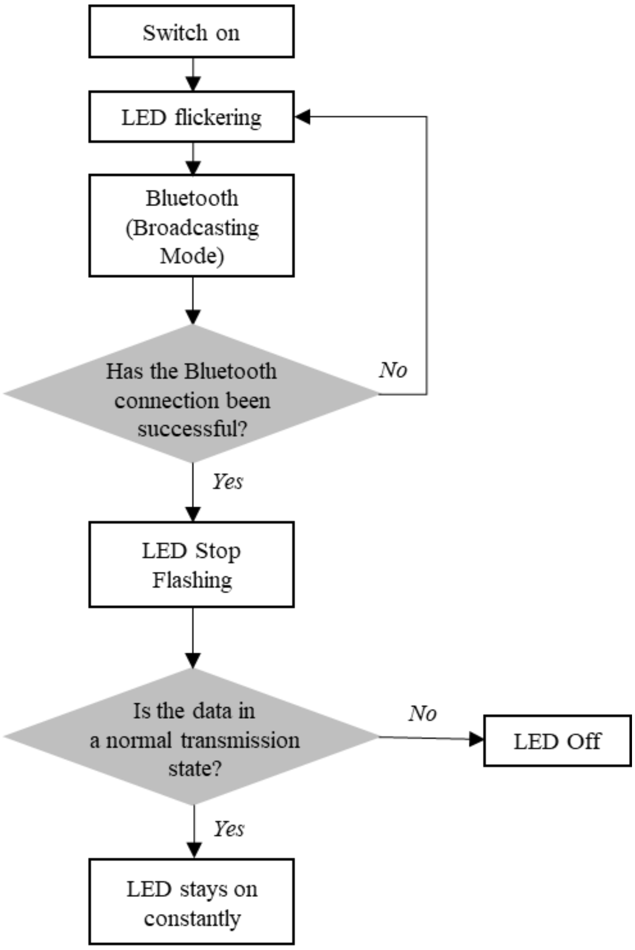

Figure S3. Program logic diagram of the LED light in the SWD system.

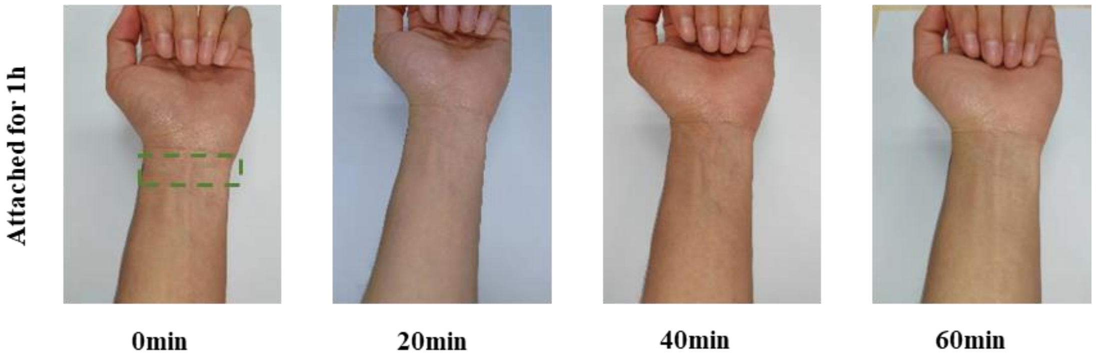

Figure S4. Optical images of the forearm after peeling off the SWD over the course of 1 hour to show no skin irritation.

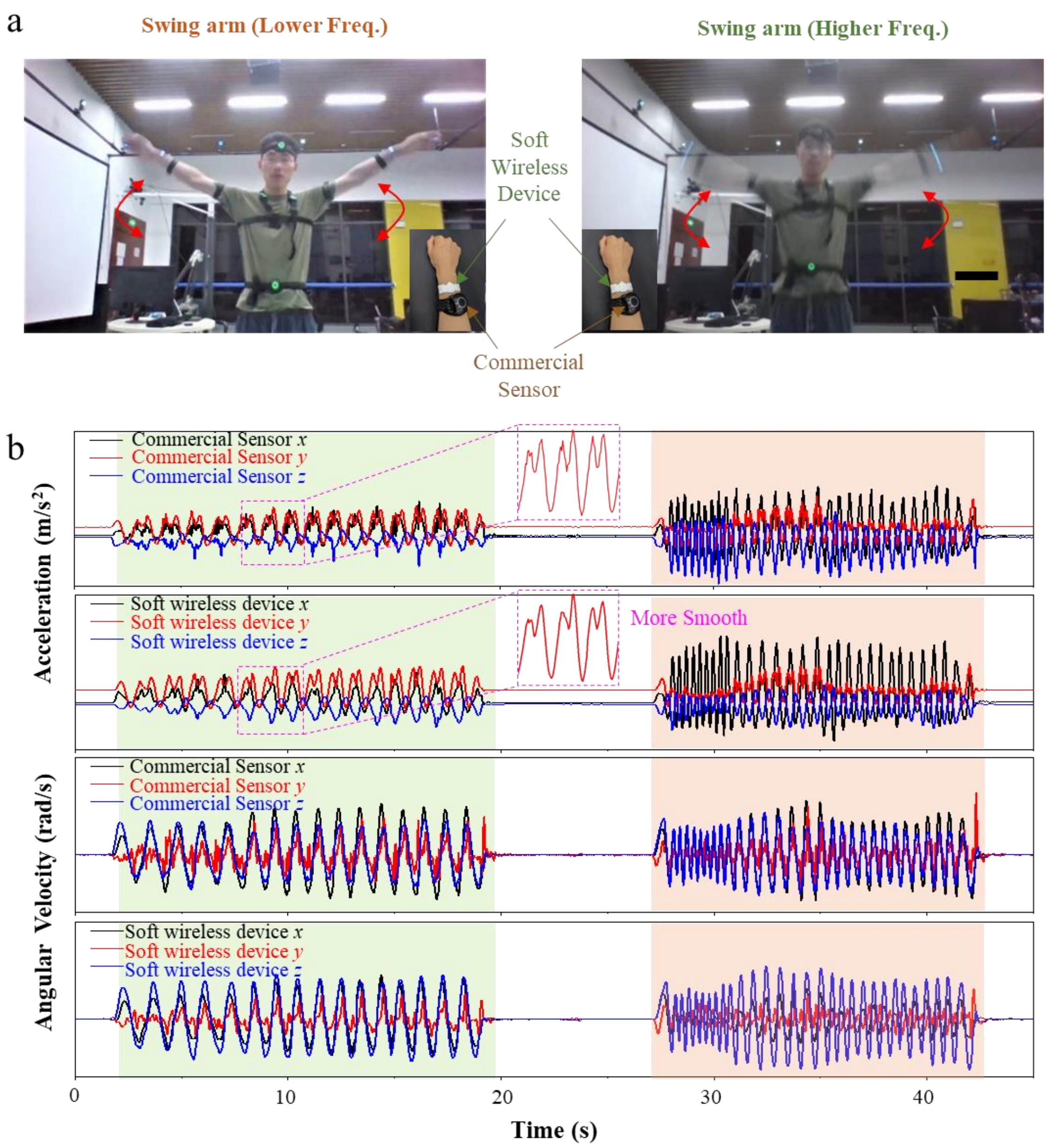

Figure S5. Comparison of high-frequency and low-frequency motion monitoring between SWD sensors and the commercial IMU device. (a) Optical images of the experimental setup with the position of the devices shown in the inset. **(b)** High-frequency and low-frequency acceleration and angular velocity obtained from SWD sensors and the commercial device.

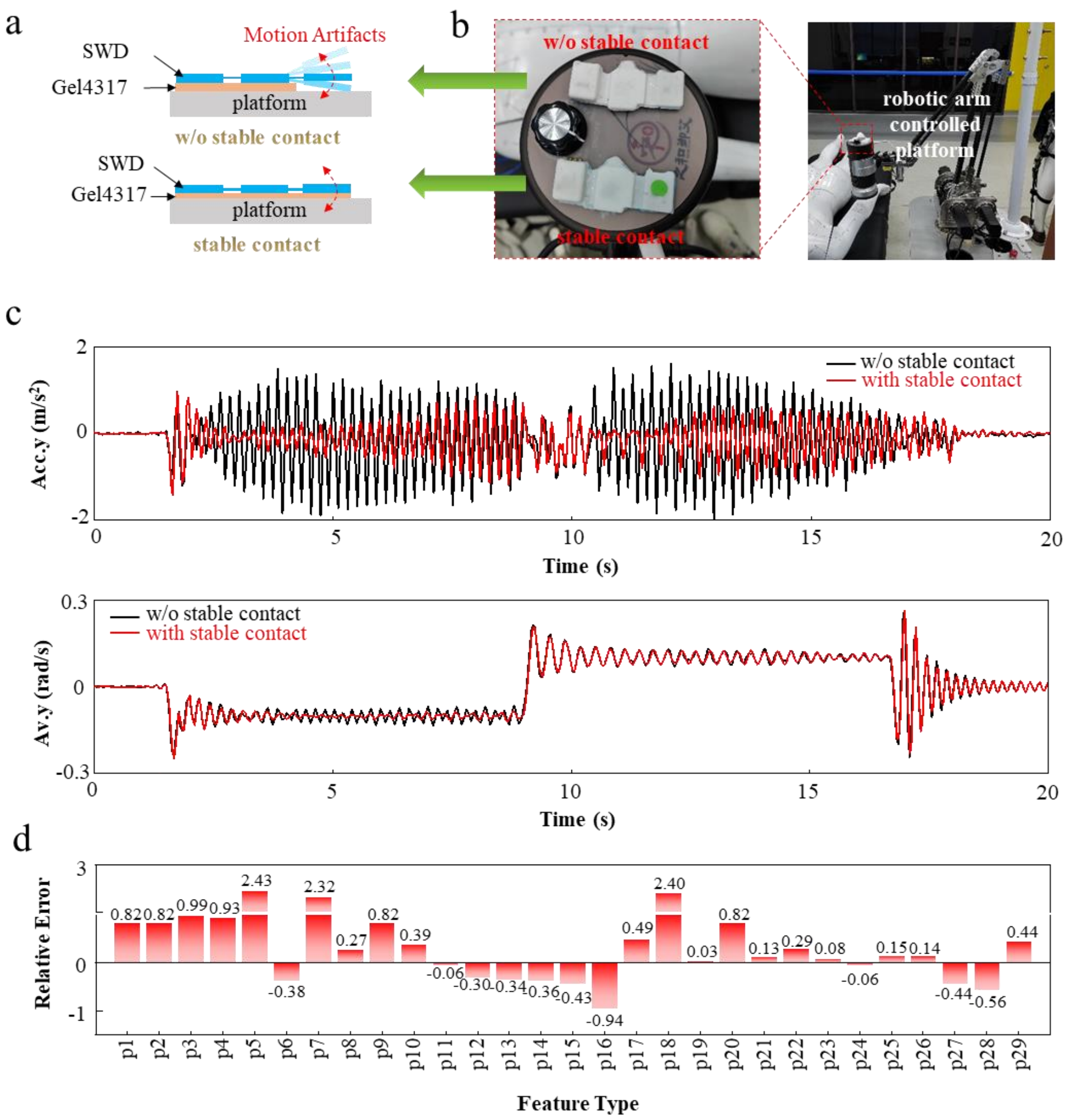

Figure S6. Comparison of motion monitoring and feature calculation between SWD sensors with and without stable/intimate contact. (a) Schematic illustration and **(b)** optical images of the experimental setup with the position of the devices shown in the inset. **(c)** Acceleration and angular velocity and **(d)** relative error of various feature values calculated from the obtained data in (**c**).

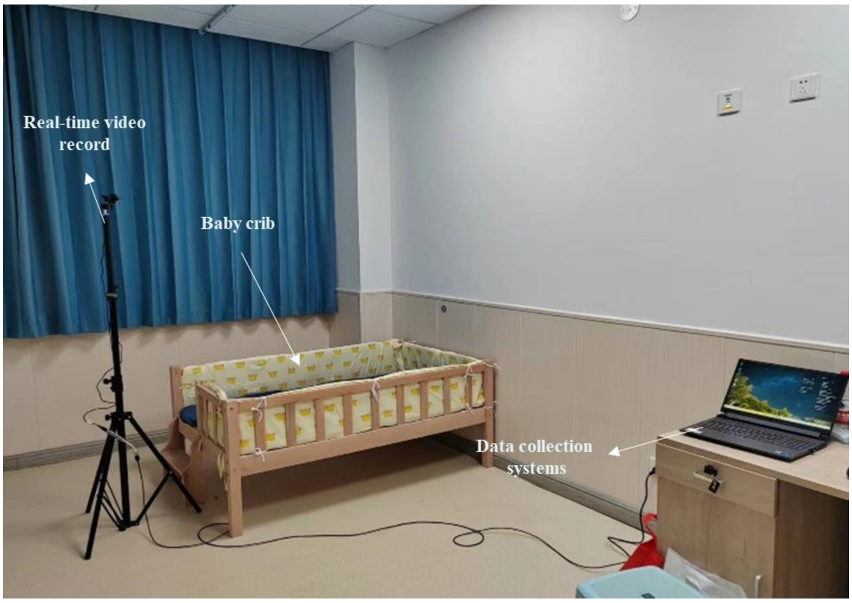

Figure S7. Optical image showing the clinical setup for collecting neonatal movement data.

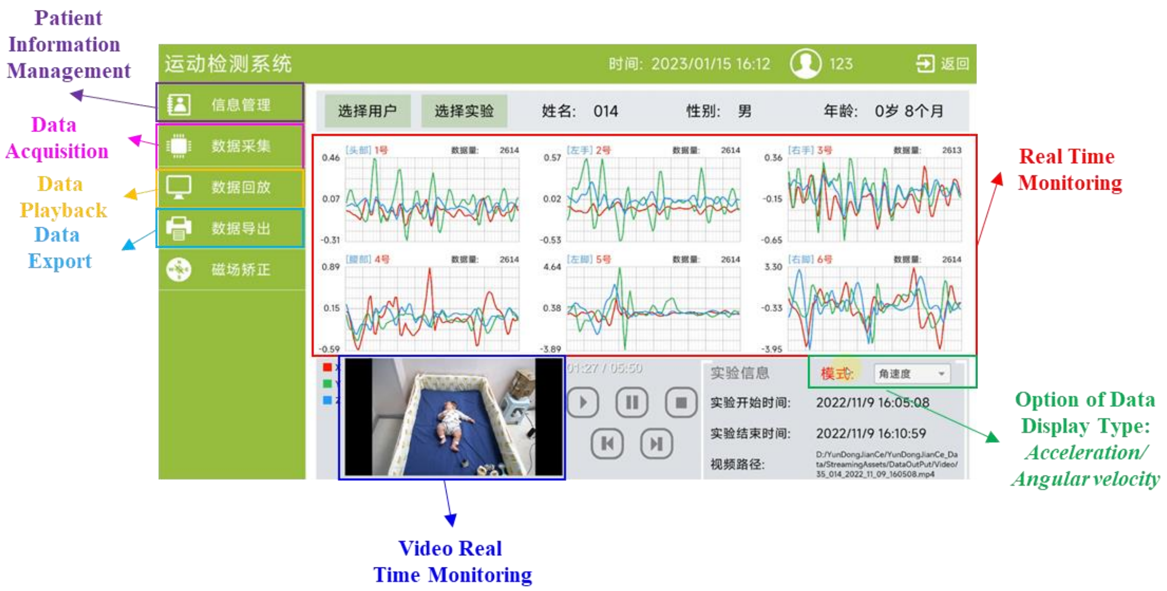

Figure S8. The screenshot of the self-developed graphics user interface.

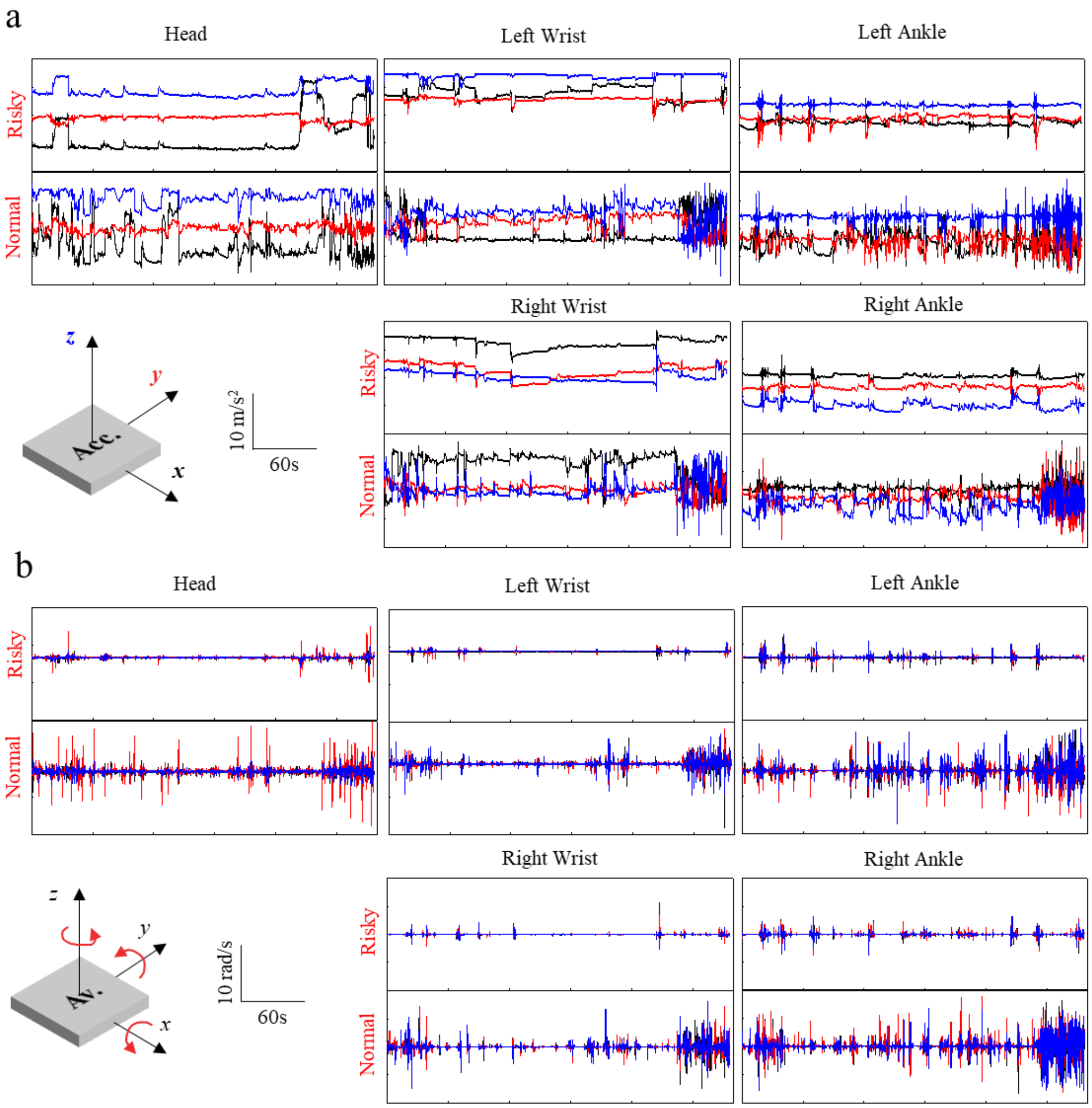

Figure S9. 2D line graphs of acceleration and angular velocity for (a) “Normal” (ID-01) and (b) “Risk” (ID-20) samples.

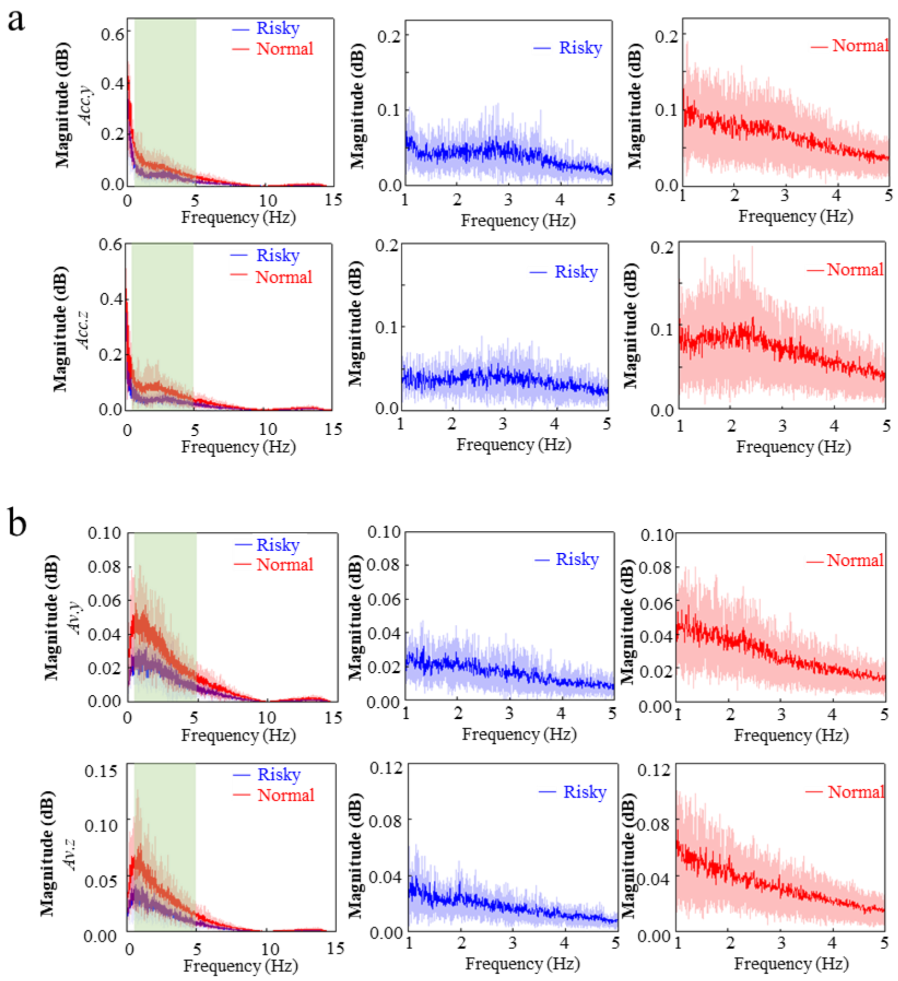

Figure S10. Power spectrum of the acceleration and angular velocity obtained from the left ankle. (a) The power spectrum of the *x*-axis (Acc.x), *y*-axis (Acc.y), and z-axis acceleration (Acc.z) with error band. **(b)** The power spectrum of the *x*-axis (Av.x), *y*-axis (Av.y), and *z*-axis angular velocity (Av.z) with error band. The red and blue lines represent the average power magnitude of all “Normal” and “Risk” samples (n = 18).

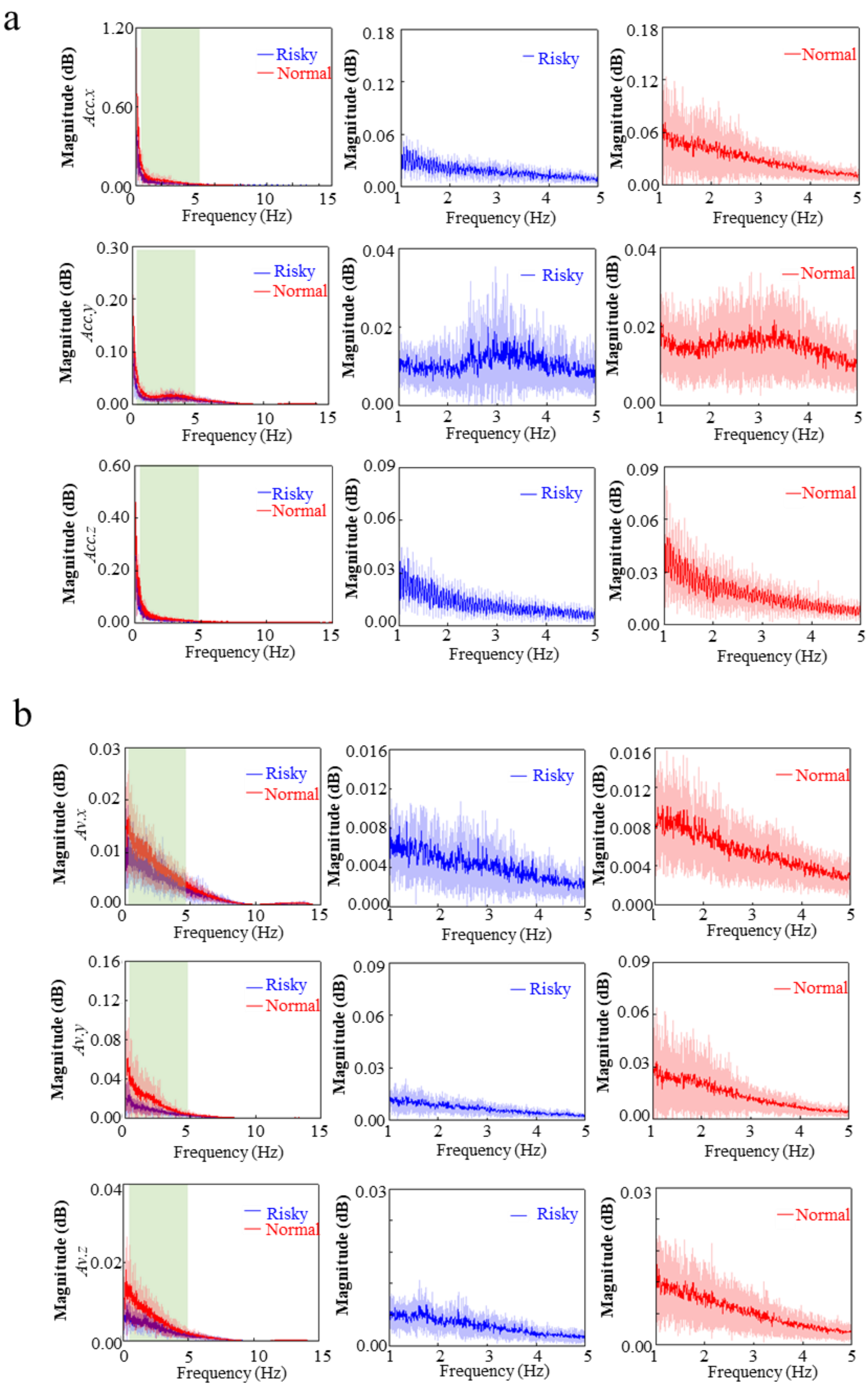

Figure S11. Power spectrum of the acceleration and angular velocity obtained from the head. (a) The power spectrum of the *x*-axis (Acc.x), *y*-axis (Acc.y), and z-axis acceleration (Acc.z) with error band. **(b)** The power spectrum of the *x*-axis (Av.x), *y*-axis (Av.y), and *z*-axis angular velocity (Av.z) with error band. The red and blue lines represent the average power magnitude of all “Normal” and “Risk” samples (n = 18).

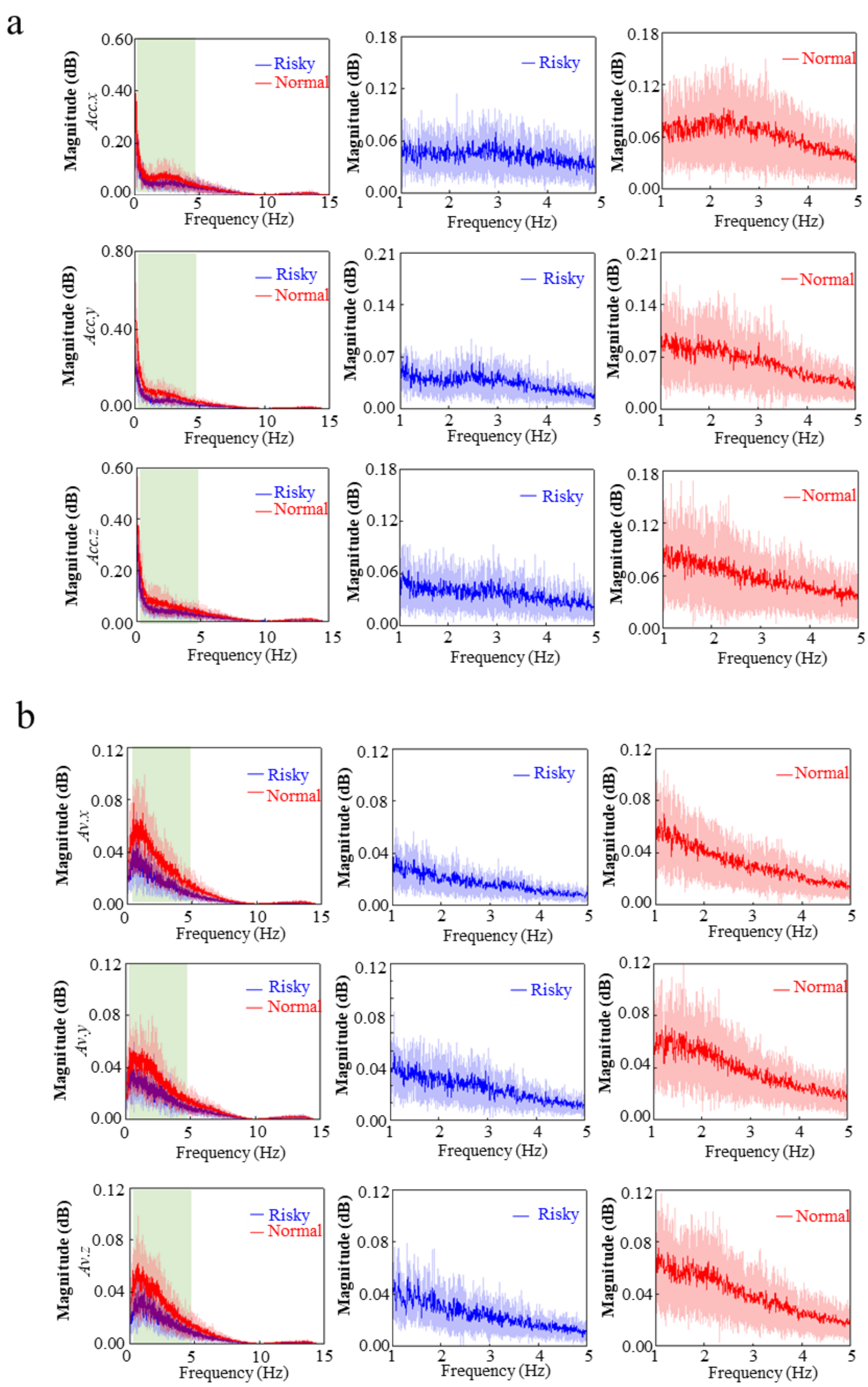

Figure S12. Power spectrum of the acceleration and angular velocity obtained from the right ankle. (a) The power spectrum of the *x*-axis (Acc.x), *y*-axis (Acc.y), and z-axis acceleration (Acc.z) with error band. **(b)** The power spectrum of the *x*-axis (Av.x), *y*-axis (Av.y), and *z*-axis angular velocity (Av.z) with error band. The red and blue lines represent the average power magnitude of all "Normal" and "Risk" samples (n = 18).

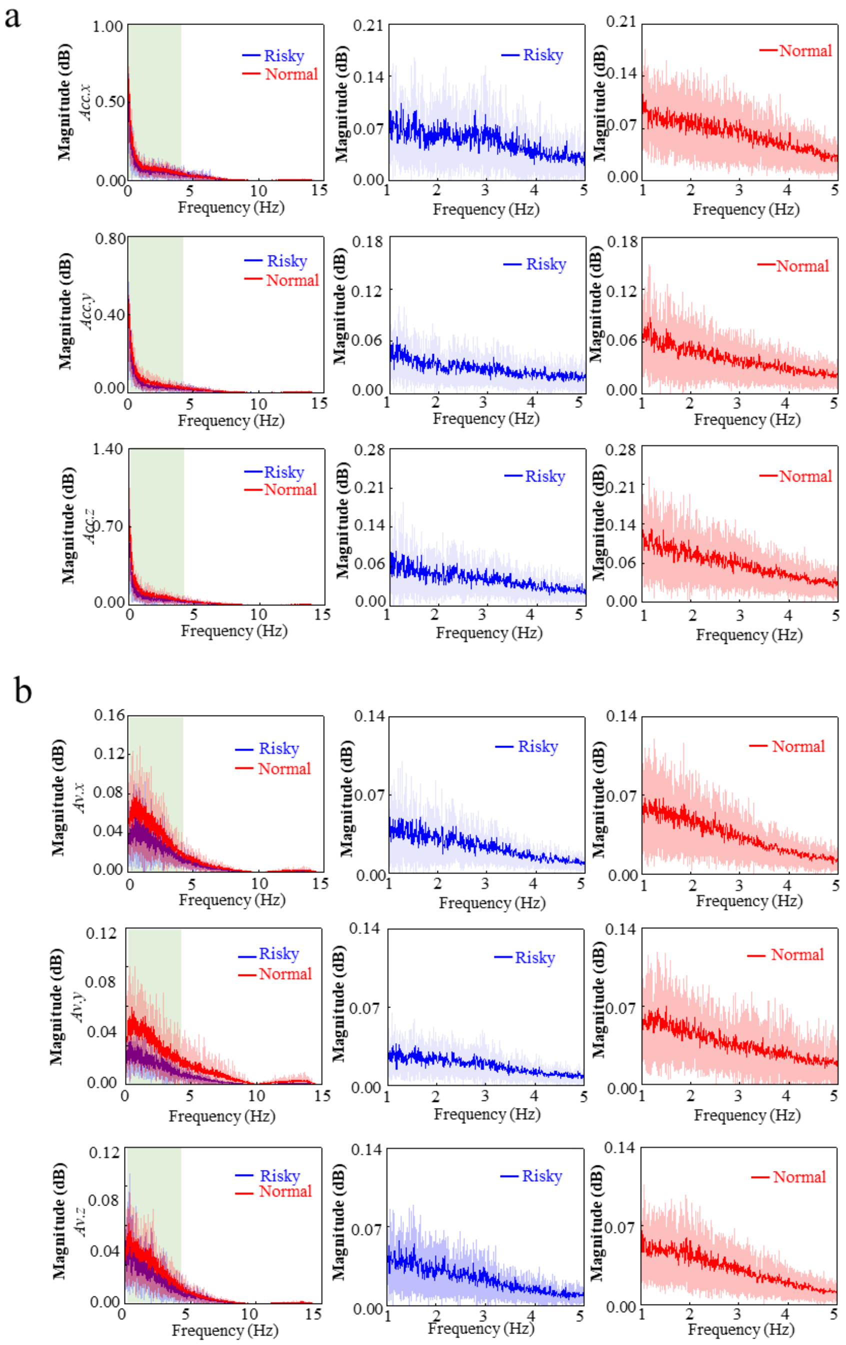

Figure S13. Power spectrum of the acceleration and angular velocity obtained from the left wrist. (a) The power spectrum of the *x*-axis (Acc.x), *y*-axis (Acc.y), and z-axis acceleration (Acc.z) with error band. **(b)** The power spectrum of the *x*-axis (Av.x), *y*-axis (Av.y), and *z*-axis angular velocity (Av.z) with error band. The red and blue lines represent the average power magnitude of all “Normal” and “Risk” samples (n = 18).

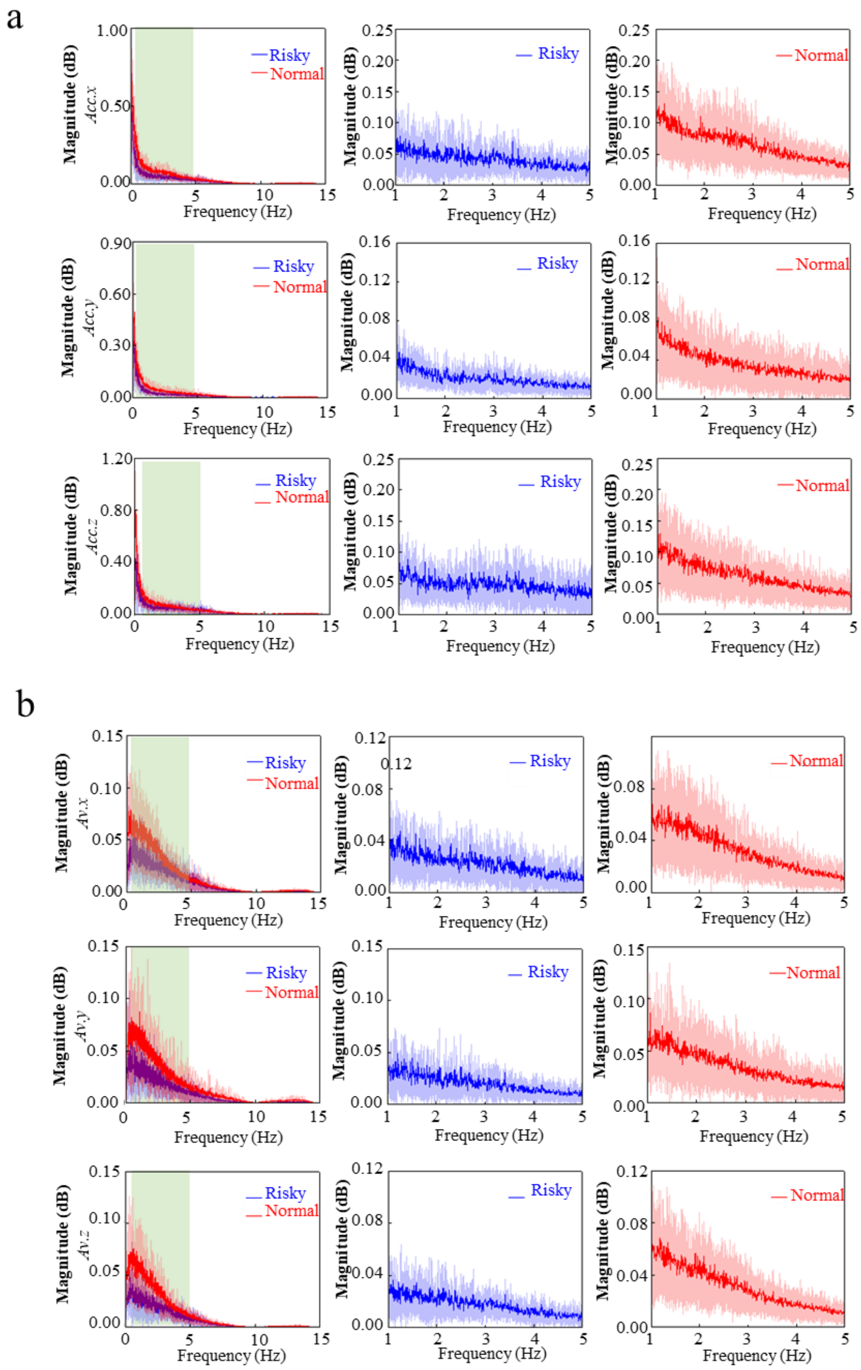

Figure S14. Power spectrum of the acceleration and angular velocity obtained from the right wrist. (a) The power spectrum of the *x*-axis (Acc.x), *y*-axis (Acc.y), and z-axis acceleration (Acc.z) with error band. **(b)** The power spectrum of the *x*-axis (Av.x), *y*-axis (Av.y), and *z*-axis angular velocity (Av.z) with error band. The red and blue lines represent the average power magnitude of all “Normal” and “Risk” samples (n = 18).

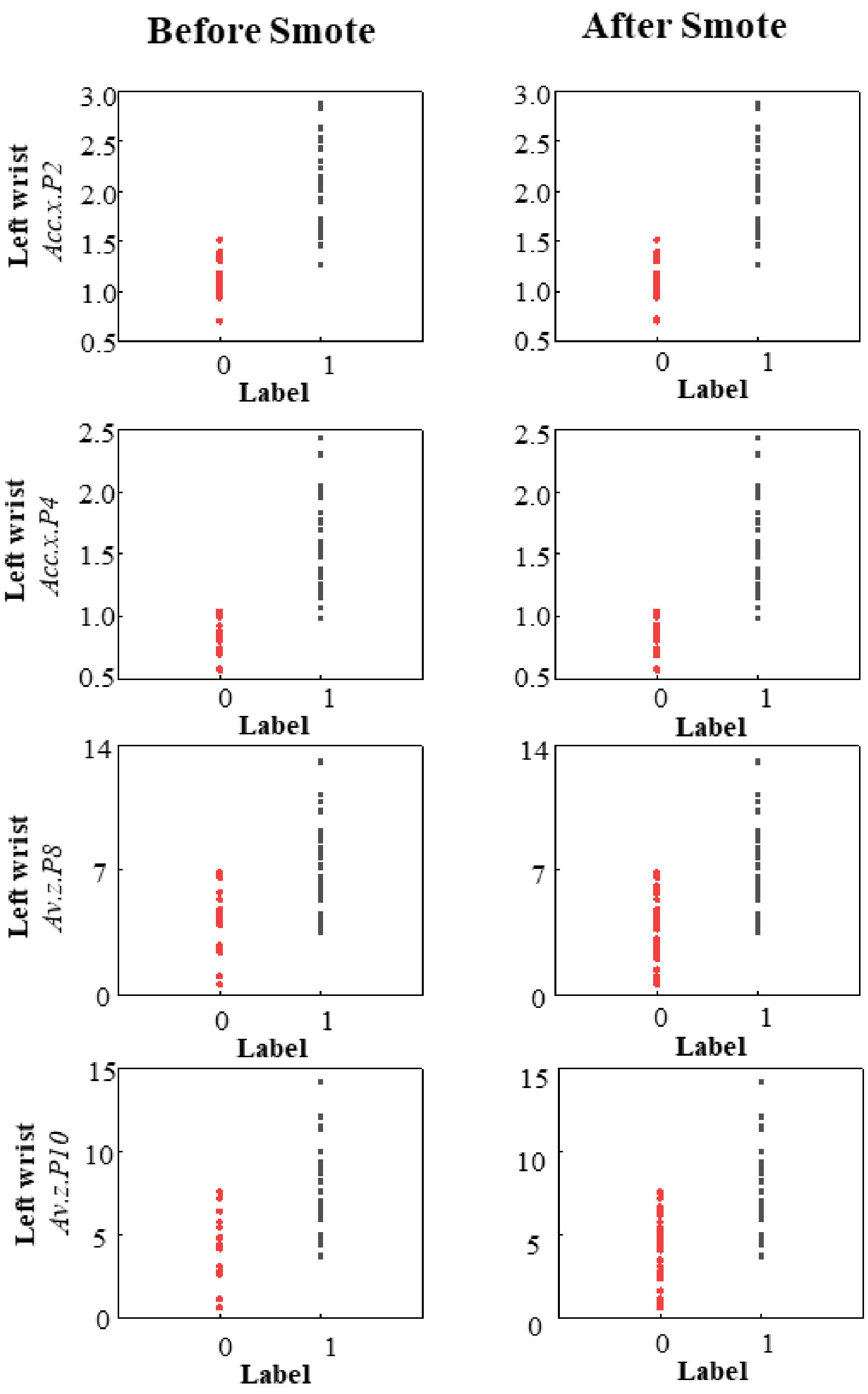

Figure S15. Distribution of features from the left wrist sensing data before (left) and after (right) SMOTE. Red/black: risk/normal samples.

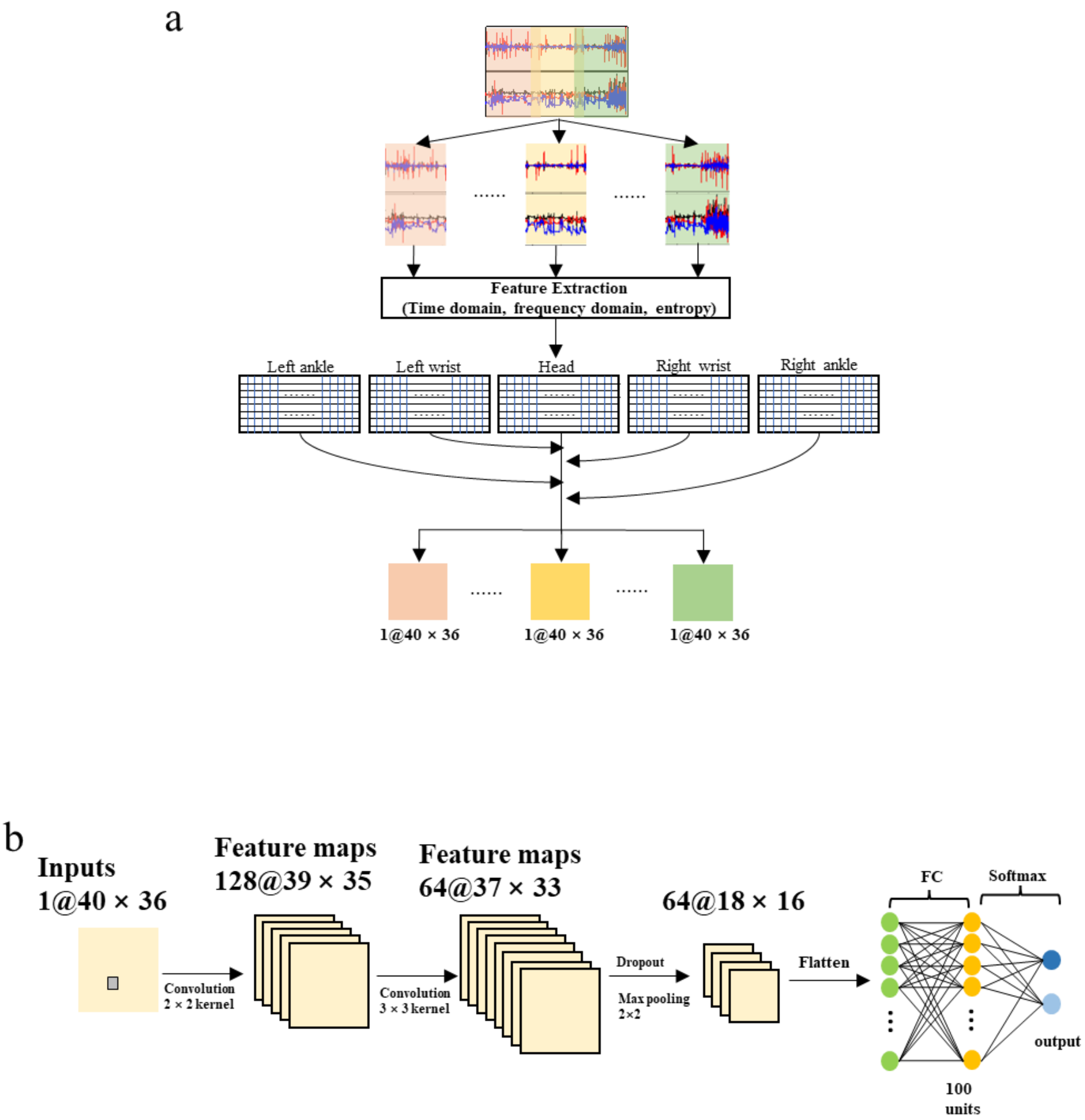

Figure S16. Diagram showing the construction of the convolutional neural network (CNN). (a) The data processing flow from subjects' motion data to 2D feature maps. **(b)** Diagram showing the structure of the convolutional network.

Table S1. Comparison between this work and other systems for infant neurodevelopmental assessment in terms of size, quality, system composition, and cost

Name	Size（mm）	Cost (USD)	Component	Automatic auxiliary evaluated function	Ref.
Magnetic Resonance Imaging (MRI) machines	~4000×2000×3000	~$440,000	main magnet, gradient field, RF coil, computer system	no	[2]
GoPro System	71×55×33	~$847	2 GoPro cameras	yes	[3]
Vicon Motion Capture System	120×80×24	$4,850	7 infrared cameras	no	[4]
Microsoft Azure Kinect	126×103×39	$399	cameras, IMU, computer system	yes	[5]
CORE	32×21×3	<$300	8 sensors (IMU)	no	[6]
**SSN-SWD**	**54×27×4**	**<$75**	**5 sensors (IMU), computer system**	**yes**	**this work**

Table S2. Cost analysis of a single SWD

Component	Description	Quantity	Unit price	Total price
2.2μF	Capacitors	1	$0.0084	$0.0084
10uF	Capacitors	3	$0.0140	$0.0420
100nF	Capacitors	6	$0.0056	$0.0336
22pF	Capacitors	1	$0.0056	$0.0056
1uF	Capacitors	1	$0.0084	$0.0084
RGB Led	NH-B1212RGBA-HF	1	$0.0420	$0.0420
PGB1010603NR	Bidirectional surface mount polymeric ESD suppressor	1	$0.0560	$0.0560
9V/0.5A	Fuse wire	1	$0.0560	$0.0560
10K	Resistors	5	$0.0042	$0.0210
100K	Resistors	2	$0.0042	$0.0082
10.8K	Resistors	1	$0.0042	$0.0042
2K	Resistors	1	$0.0042	$0.0042
1K	Resistors	3	$0.0042	$0.0042
ND08	Bluetooth module	1	$3.9269	$3.9269
TLV70028DCKR	Linear voltage regulators	1	$0.7012	$0.7012
MPU9250	Motion chip	1	$4.9079	$4.9079
BQ24040	Linear battery charger	1	$0.4487	$0.4487
VKD233DR	Single button touch detection chip	1	$0.1122	$0.1122
FPC Fabrication	Double-layer	1	$2.6292	$2.6292
Ecoflex0030	Materials for shell preparation	1	~$0.5047	~$0.5047
Slice Pig	Shell pigments	1	~$0.0701	~$0.0701
Total				~$13.5947

Table S3. Subject information with expert ratings

ID	Gender	Postmenstrual Age (weeks)	Goal	Binary Classification	Three-label Classification
**01**	Female	12	**F+**	Normal	Normal
**02**	Male	16	**F+**	Normal	Normal
**03**	Female	12	**F±**	Risky	Low risk
**04**	Female	13	**F+**	Normal	Normal
**05**	Female	18	**F+**	Normal	Normal
**06**	Female	9	**F+**	Normal	Normal
**07**	Male	17	**F±**	Normal	Low risk
**08**	Female	17	**F+**	Normal	Normal
**09**	Male	11	**F-**	Risky	High risk
**10**	Male	Invalid Data			
**11**	Female	11	**F+**	Normal	Normal
**12**	Male	Invalid Data			
**13**	Male	13	**F+**	Normal	Normal
**14**	Male	Invalid Data			
**15**	Male	10	**F+**	Normal	Normal
**16**	Male	4	**N**	Normal	Normal
**17**	Male	10	**F+**	Normal	Normal
**18**	Female	17	**F±**	Risky	Low risk
**19**	Female	11	**F+**	Normal	Normal
**20**	Male	6	**PR**	Risky	High risk
**21**	Male	Invalid Data			
**22**	Male	Invalid Data			
**23**	Male	21	**F-**	Risky	High risk

Note: "F+": normal general movements; "F±": abnormal general movements; "F-": slight absence of general movements; "PR": poor repertoire general movements, which is a type of the absence of general movements.

Table S4. List of the 36 features with corresponding abbreviations

ID	Feature	Type
P1	Mean	*Time*
P2	Root mean square(RMS)	*Time*
P3	Square root magnitude	*Time*
P4	Absolute average	*Time*
P5	Skewness	*Time*
P6	Kurtosis	*Time*
P7	Variance	*Time*
P8	Max	*Time*
P9	Min	*Time*
P10	Peak-to-peak	*Time*
P11	Form factor	*Time*
P12	Crest factor	*Time*
P13	Impulse factor	*Time*
P14	Margin factor	*Time*
P15	Skewness factor	*Time*
P16	Kurtosis factor	*Time*
P17	Mean frequency	*Frequency*
P18	Standard deviation of the frequency	*Frequency*
P19	The degree of dispersion or concentration of the spectrum	*Frequency*
P20	DC component	*Frequency*
P21	Frequency center	*Frequency*
P22	The degree of dispersion or concentration of the spectrum	*Frequency*
P23	RMS frequency	*Frequency*
P24	Indicates a change in the position of the main frequency band	*Frequency*
P25	Indicates a change in the position of the main frequency band	*Frequency*
P26	The degree of dispersion or concentration of the spectrum	*Frequency*
P27	The degree of dispersion or concentration of the spectrum	*Frequency*
P28	The degree of dispersion or concentration of the spectrum	*Frequency*
P29	The degree of dispersion or concentration of the spectrum	*Frequency*
P30	Power spectrum entropy	*Entropy*
P31	Energy entropy	*Entropy*
P32	Approximate entropy	*Entropy*
P33	Sample entropy	*Entropy*
P34	Fuzzy entropy	*Entropy*
P35	Permutation entropy	*Entropy*
P36	Envelope entropy	*Entropy*

Table S5. Classification results from different machine learning models using all features without SMOTE

Models	Accuracy	Precision	Recall	F-Score
**KNN**	0.9632	0.9815	0.9695	0.9725
**DT**	0.9226	0.9563	0.9355	0.9412
**RF**	0.9208	0.9719	0.9140	0.9376
**ET**	0.9719	0.9744	0.9855	0.9789
**GNB**	0.9788	0.9718	1.0000	0.9841
**MNB**	0.9333	0.9997	0.9034	0.9461
**SVM**	0.9156	0.8957	1.0000	0.9403
**AdaBoost**	0.9293	0.9620	0.9369	0.9459
**LR**	0.9980	0.9976	1.0000	0.9984

Table S6. Classification results from different machine learning models using all features with SMOTE

Models	Accuracy	Precision	Recall	F-Score
**KNN**	0.9644	1.0000	0.9320	0.9614
**DT**	0.9582	0.9803	0.9392	0.9558
**RF**	0.9515	0.9896	0.9155	0.9476
**ET**	0.9867	0.9941	0.9800	0.9860
**GNB**	0.9926	0.9871	1.0000	0.9927
**MNB**	0.9960	0.9989	0.9926	0.9954
**SVM**	0.8889	0.9917	0.8047	0.8770
**AdaBoost**	0.9606	0.9864	0.9374	0.9582
**LR**	1.0000	1.0000	0.9993	1.0000

Table S7. Dimensions of feature matrices for different models to achieve the highest accuracy

Models	Dimensions of feature matrices	Highest accuracy
**KNN**	40	0.9774
**DT**	20	0.9640
**RF**	40	0.9600
**ET**	40	0.9868
**GNB**	50	0.9928
**MNB**	60	0.9960
**SVM**	10	0.9146
**AdaBoost**	10	0.9667
**LR**	50	1.0000

Supplementary videos

Movie S1 (.mp4 format). Performance of SWD during stretching and twisting.
Movie S2 (.mp4 format). Simulation of SWD folding.

References:

[1]C. Romano, E. Schena, D. Formica, C. Massaroni, *Biosensors (Basel)* **2022**, *12* (10), 834. https://doi.org/10.3390/bios12100834.

[2]A. J. Spittle, R. N. Boyd, T. E. Inder, L. W. Doyle, *Pediatrics* **2009**, *123* (2), 512. https://doi.org/10.1542/peds.2008-0590.

[3]C. Chambers, N. Seethapathi, R. Saluja, H. Loeb, S. R. Pierce, D. K. Bogen, L. Prosser, M. J. Johnson, K. P. Kording, *IEEE Transactions on Neural Systems and Rehabilitation Engineering* **2020**, *28* (11), 2431. https://doi.org/10.1109/TNSRE.2020.3029121.

[4]L. Meinecke, N. Breitbach-Faller, C. Bartz, R. Damen, G. Rau, C. Disselhorst-Klug, *Human Movement Science* **2006**, *25* (2), 125. https://doi.org/10.1016/j.humov.2005.09.012.

[5]Q. Wu, G. Xu, F. Wei, L. Chen, S. Zhang, *IEEE Access* **2021**, *9*, 42314. https://doi.org/10.1109/ACCESS.2021.3066148.

[6]H. Jeong, S. S. Kwak, S. Sohn, J. Y. Lee, Y. J. Lee, M. K. O'Brien, Y. Park, R. Avila, J.-T. Kim, J.-Y. Yoo, M. Irie, H. Jang, W. Ouyang, N. Shawen, Y. J. Kang, S. S. Kim, A. Tzavelis, K. Lee, R. A. Andersen, Y. Huang, A. Jayaraman, M. M. Davis, T. Shanley, L. S. Wakschlag, S. Krogh-Jespersen, S. Xu, S. W. Ryan, R. L. Lieber, J. A. Rogers, *Proceedings of the National Academy of Sciences* **2021**, *118* (43), e2104925118. https://doi.org/10.1073/pnas.2104925118.
